# Genome Capture Sequencing Selectively Enriches Bacterial DNA and Enables Genome-Wide Measurement of Intrastrain Genetic Diversity in Human Infections

**DOI:** 10.1128/mbio.01424-22

**Published:** 2022-09-19

**Authors:** Hillary S. Hayden, Snehal Joshi, Matthew C. Radey, Anh T. Vo, Cara Forsberg, Sarah J. Morgan, Adam Waalkes, Elizabeth A. Holmes, Sara M. Klee, Mary J. Emond, Pradeep K. Singh, Stephen J. Salipante

**Affiliations:** a Department of Microbiology, University of Washington School of Medicine, Seattle, Washington, USA; b Department of Genome Sciences, University of Washington School of Medicine, Seattle, Washington, USA; c Department of Laboratory Medicine and Pathology, University of Washington School of Medicine, Seattle, Washington, USA; d Department of Biostatistics, University of Washington, Seattle, Washington, USA; Marquette University; University of Pittsburgh School of Medicine

**Keywords:** evolution, genetic diversity, genomics, infection, microbiology, targeted enrichment

## Abstract

Within-host evolution produces genetic diversity in bacterial strains that cause chronic human infections. However, the lack of facile methods to measure bacterial allelic variation in clinical samples has limited understanding of intrastrain diversity’s effects on disease. Here, we report a new method termed genome capture sequencing (GenCap-Seq) in which users inexpensively make hybridization probes from genomic DNA or PCR amplicons to selectively enrich and sequence targeted bacterial DNA from clinical samples containing abundant human or nontarget bacterial DNA. GenCap-Seq enables accurate measurement of allele frequencies over targeted regions and is scalable from specific genes to entire genomes, including the strain-specific accessory genome. The method is effective with samples in which target DNA is rare and inhibitory and DNA-degrading substances are abundant, including human sputum and feces. In proof-of-principle experiments, we used GenCap-Seq to investigate the responses of diversified Pseudomonas aeruginosa populations chronically infecting the lungs of people with cystic fibrosis to *in vivo* antibiotic exposure, and we found that treatment consistently reduced intrastrain genomic diversity. In addition, analysis of gene-level allele frequency changes suggested that some genes without conventional resistance functions may be important for bacterial fitness during *in vivo* antibiotic exposure. GenCap-Seq’s ability to scalably enrich targeted bacterial DNA from complex samples will enable studies on the effects of intrastrain and intraspecies diversity in human infectious disease.

## INTRODUCTION

While progress against acute bacterial infections has been remarkable, the understanding and treatment of chronic infections have lagged. One distinguishing characteristic of chronic infections is that infecting strains often evolve genetic diversity, sometimes to a vast extent (1). Multiple features of chronic infections promote diversification of infecting strains, including large bacterial population sizes, long infection duration, heterogenous selective pressures, and geographic isolation of subpopulations in different organ regions (2). The chronic Pseudomonas aeruginosa lung infections that afflict people with cystic fibrosis (CF) are a prime example. During CF lung infections, P. aeruginosa strains evolve into populations of genetic variants that can differ markedly in stress and antibiotic resistance, virulence, nutrient utilization, and other key phenotypes (3). P. aeruginosa in chronically infected wounds and sinuses (4), *Burkholderia* strains causing CF infections (5), Helicobacter pylori infecting stomach mucosa (6), and Mycobacterium tuberculosis strains (7) have also been shown to evolve diversity during human infection.

Theory predicts that intrastrain genetic diversity could enhance bacterial persistence and affect disease manifestations and treatment responses. For example, variants with high tolerance to stress or antibiotics could increase in abundance during periods of treatment or immune activation and compensate for the loss of susceptible variants (8). In addition, changes in the abundance of variants with increased invasive potential could mediate shifts between disease quiescence and disease flares that occur frequently in chronic infections (9). Complementary variants could also enable cooperative interactions (10). For instance, toxic variants could disable immune responses and increase nutrient availability to the benefit of other clonally related subpopulations.

Measuring intrastrain genetic variation in clinical samples is challenging, and existing methods have limitations. For example, studying cultured isolates requires sequencing many colonies for accurate sampling, and populations may be skewed by culture. Direct sequencing of DNA from clinical samples obviates culture but can be limited by the vast excess of nontarget DNA, as host DNA dominates sputum (11), skin (12), and blood and nasal specimens (13), even when pathogen counts are high. Similarly, individual species or strains typically comprise only a fraction of total biomass of fecal samples (14), even when most of the DNA present is bacterial. While innovative approaches have been developed to address these problems (see Discussion), new methods that are inexpensive and scalable are needed to enable genome-wide measurements of genetic variants in targeted organisms.

Here, we describe a new method termed GenCap-Seq (genome capture sequencing) in which hybridization capture probes are generated by users from the genomic DNA of cultured isolates or PCR amplicons in order to enrich targeted bacterial DNA from complex samples. Enriched DNA is then sequenced to measure the abundance of genetic variants present over the entire genome or designated regions. We characterized the performance of this approach using synthetic samples and clinical specimens, and we performed exploratory studies to investigate changes in bacterial intrastrain genetic diversity in response to antibiotic treatment.

## RESULTS

### Technical approach.

**FIG 1:**
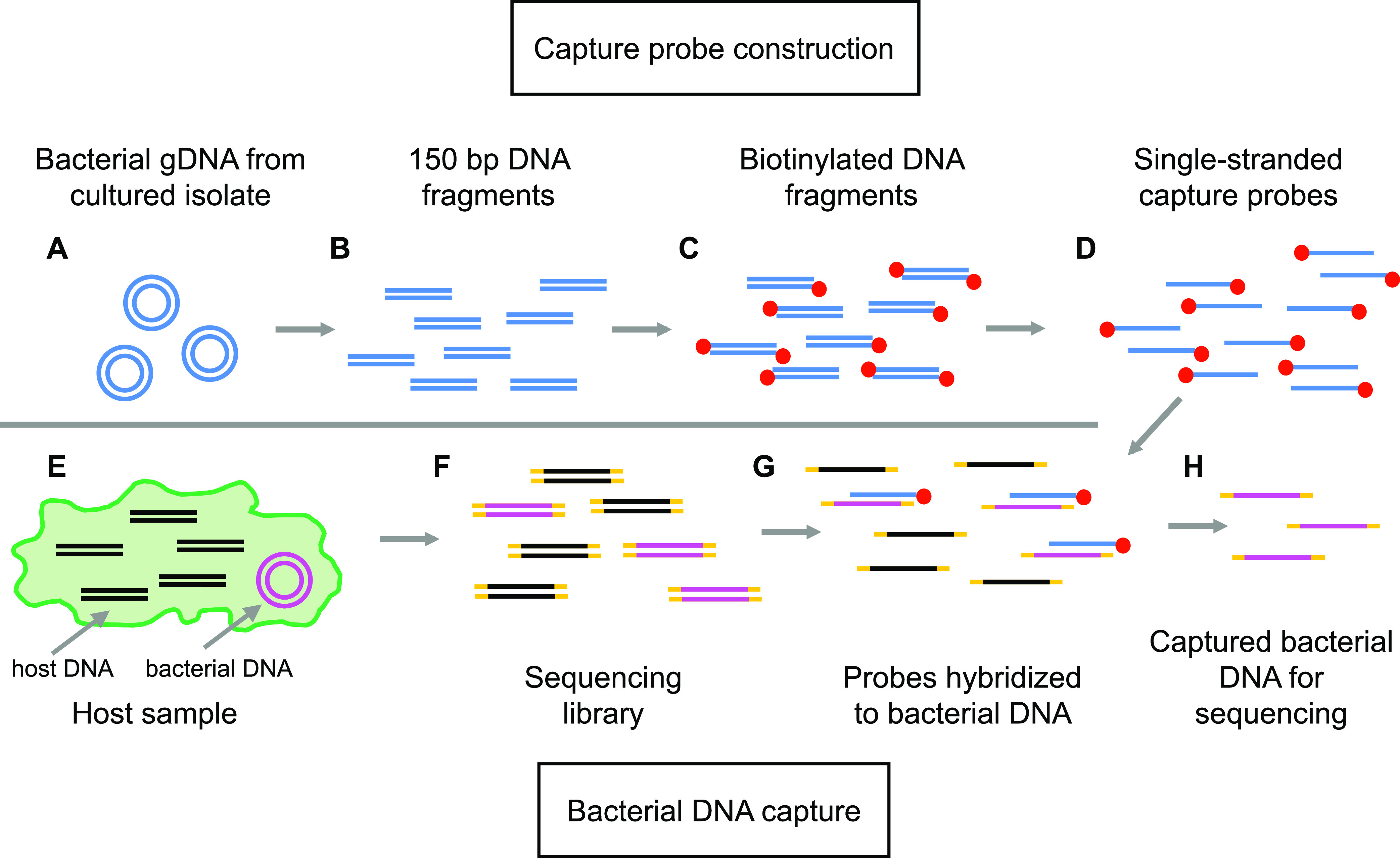
The GenCap-Seq method for whole-genome enrichment. (A and B) To generate capture probes, bacterial genomic DNA (gDNA) extracted from a representative cultured isolate (A) is sheared to 150-bp fragments (B). (C) Fragments are end-repaired and biotinylated. (D) Biotinylated fragments are denatured to produce single-stranded, biotinylated capture probes, and nonbiotinylated fragments are removed. (E) In primary specimens, DNA from both undesired organisms (black lines) and microbes (pink circles) are present. (F) Total DNA is extracted, fragmented, and joined to sequencing adapters to construct a sequencing library. (G) Biotinylated capture probes hybridize to denatured library molecules, containing bacterial DNA. (H) Hybridized library molecules are captured with streptavidin beads, PCR amplified, and sequenced.

GenCap-Seq methods are illustrated in Fig. 1, and detailed protocols are provided in the supplemental material (see Text S1). Briefly, capture probes are manufactured in the user’s laboratory from the DNA of interest. Genomic DNA is used if the application requires whole-genome variant measurements, and PCR amplicons are used if specific regions are targeted. Probes are made by mechanically shearing DNA to ~150-bp lengths, followed by enzymatic biotinylation using terminal transferase and single-strand denaturation (Fig. 1A to D). Probes are then hybridized to a sequencing library prepared from the sample to be interrogated. Library DNA molecules that are complementary to probes are enriched using paramagnetic streptavidin-coated beads, amplified with minimal PCR cycles, and sequenced to generate reads from targeted DNA (Fig. 1E to H).

10.1128/mbio.01424-22.2 TEXT S1 Extended methods, including protocols for whole-genome probe preparation and for probe hybridization and enrichment. Download Text S1, DOCX file, 0.04 MB. Copyright © 2022 Hayden et al. 2022 Hayden et al. https://creativecommons.org/licenses/by/4.0/ This content is distributed under the terms of the Creative Commons Attribution 4.0 International license.

For variant analysis, sequence reads can be aligned to a reference or subject-specific strain. Because sample DNA is randomly fragmented for sequencing library preparation, DNA from individual bacterial chromosomes will have unique 5′- and 3′-end positions. After removal of PCR duplicates resulting from posthybridization amplification, the number of chromosomes interrogated at specific genomic positions can therefore be estimated as the count of sequence reads with unique 5′ and 3′ ends. This capability contrasts with those of PCR-based enrichment strategies (15), which result in products having identical starts and ends. In addition to variant calling, the enriched sequence reads can be used for *de novo* genome assembly.

### Synthetic samples to evaluate GenCap-Seq performance.

The performance of GenCap-Seq depends primarily on two capabilities: (i) its efficacy in enriching low-prevalence target DNA from complex mixtures, and (ii) the accuracy with which captured DNA sequences report nucleotide variation present in the sample. To evaluate these capabilities, we created test samples composed of DNA from two P. aeruginosa strains: the reference strain PAO1 and the distantly related PACS2 strain. These strains differ at approximately 28,000 positions across ~6 MB of their shared genome sequence. DNA extracted independently from the two strains was combined in three proportions (PAO1 to PACS2 at 80:20, 90:10, and 95:05) and then diluted to 2% total P. aeruginosa abundance in a background of 98% human DNA. Our use of technical replicates for these three synthetic samples, and the fact that PAO1 and PACS2 differed at many genomic loci, enabled us to assess GenCap-Seq’s capacity for target DNA enrichment and to simultaneously make many independent measurements of allele frequency across the entire genome using these materials.

### GenCap-Seq using whole-genome probes enriches bacterial genomes in synthetic samples.

**FIG 2:**
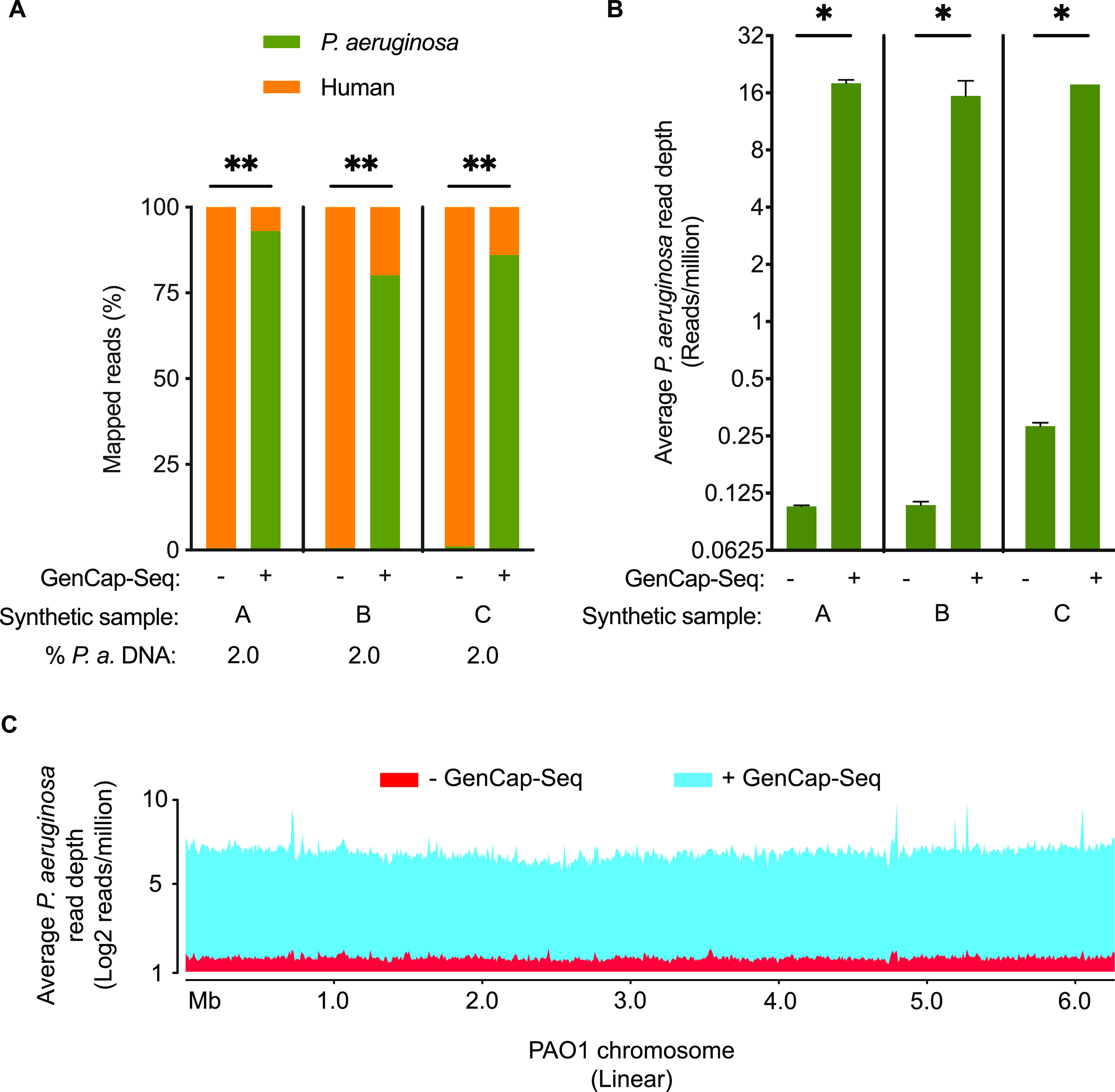
GenCap-Seq using whole-genome probes enriches for bacterial DNA in synthetic samples dominated by human DNA. (A) Percentages of sequence reads mapped to P. aeruginosa without (−) or with (+) enrichment by GenCap-Seq in three independent synthetic samples (A, B, and C), each composed of 2% total P. aeruginosa and 98% human DNA. **, *P* < 0.01 by two-tailed paired *t* test. Stacked bars indicate mean percentages of sequence reads, from 2 (−) or 3 (+) technical replicates of each synthetic sample, that align to P. aeruginosa (green) or human (orange). (B) Average read depth across the P. aeruginosa genome per million reads sequenced without (−) or with (+) GenCap-Seq enrichment for the replicates examined in panel A. *, *P* < 0.05 by two-tailed paired *t* test. (C) Average read depth per million reads sequenced that align to P. aeruginosa without (red) and with (blue) enrichment by GenCap-Seq across the 6.3 Mbp PAO1 reference genome. Data are integrated from all replicates examined in panel A.

We first tested the method’s ability to enrich target DNA using probes made from PAO1 genomic DNA. GenCap-Seq was performed on three technical replicates for each of the three independently produced synthetic samples (9 total replicates, each containing 2% P. aeruginosa DNA and 98% human DNA), and the proportion of sequence reads mapping to human and P. aeruginosa genomes was measured after removal of PCR duplicates. Conventional shotgun sequencing of two replicates per sample (6 total replicates) showed that an average of 0.7% (range, 0.5 to 1.0%) of reads mapped to P. aeruginosa (Fig. 2A), resulting in an average P. aeruginosa read depth of 0.2 (range, 0.1 to 0.3) per million sequence reads (Fig. 2B). The difference between the intended input P. aeruginosa DNA concentration (2%) and reads mapping to that genome (0.7%) likely reflected a combination of sequencing bias favoring DNA with lower (human) over higher (P. aeruginosa) G+C content and error inherent to DNA quantification and pipetting during sample preparation.

After GenCap-Seq, P. aeruginosa reads compromised an average of 86% of total (range, 80% to 93%; *P* < 0.01 two-tailed paired *t* test compared to shotgun sequencing) (Fig. 2A), yielding an average read depth of 17 (range, 12 to 19) per million sequence reads (*P* < 0.05 two-tailed paired *t* test compared to shotgun sequencing) (Fig. 2B). Read depth was consistently distributed over the entire genome (Fig. 2C).

### GenCap-Seq can enrich specific genes.

Some applications benefit from high-depth interrogation of variants occurring in specific genes, rather than lower-depth surveys of variation present across the entire genome. For example, increases in the abundance in P. aeruginosa virulence gene variants have been associated with lung function decline in CF (9), and allele frequency changes in genes affecting nutrient acquisition, drug resistance, stress tolerance, and other functions could similarly affect outcomes. Using the synthetic samples, we tested a gene-specific targeting approach by performing GenCap-Seq with probes prepared from pooled PCR amplicons of 17 P. aeruginosa pathogenicity genes distributed throughout the PAO1 genome (see Materials and Methods).

**FIG 3:**
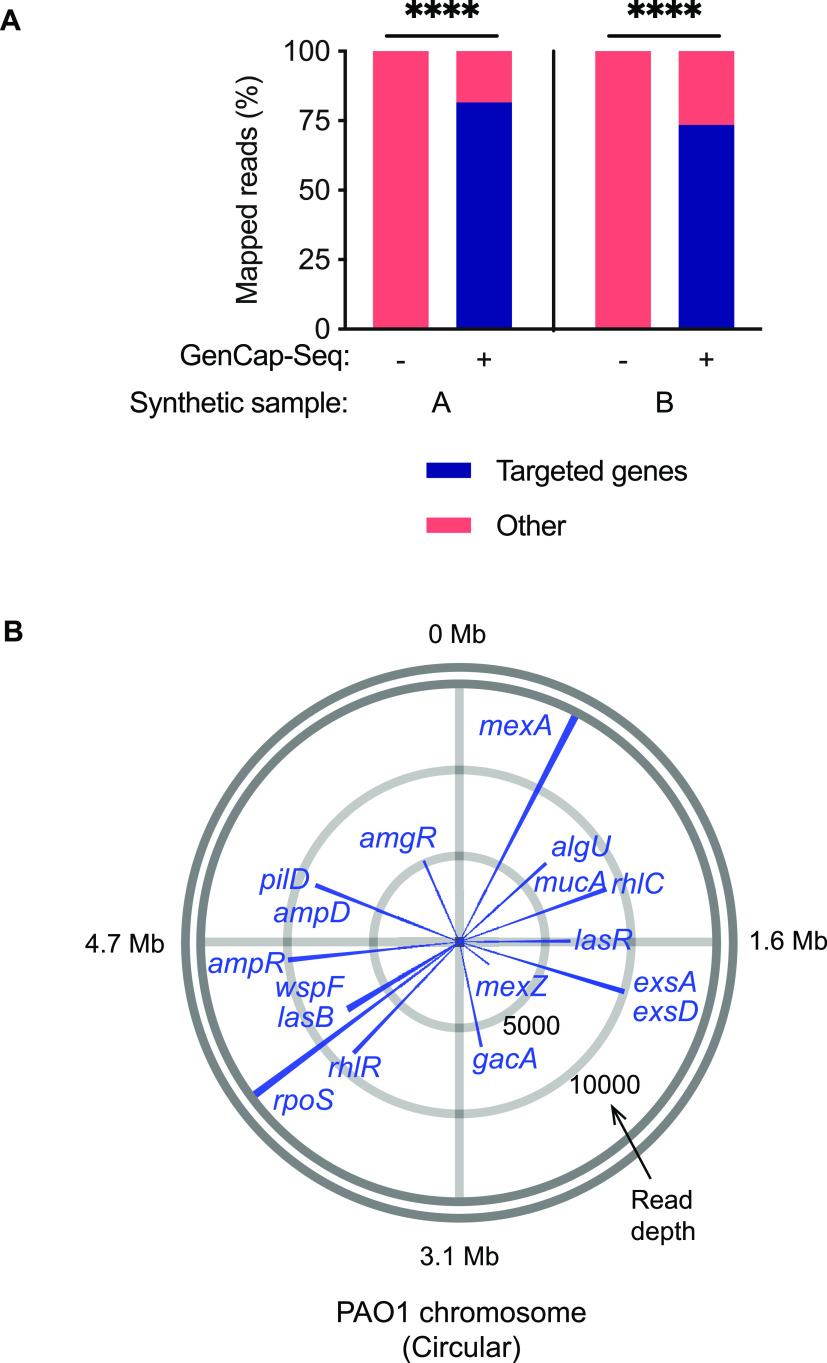
GenCap-Seq using gene-specific probes selectively enriched 17 P. aeruginosa genes. (A) Percentages of sequence reads mapped to P. aeruginosa without (−) or with (+) GenCap-Seq enrichment for 17 targeted genes in single replicates of two synthetic samples (samples A and B), each composed of 2% total P. aeruginosa and 98% human DNA. ****, *P* < 0.0001 by two-tailed paired *t* test. Stacked bars indicate percentages of sequence reads that align to the targeted genes (blue) or nontargeted DNA (salmon). (B) Representative circle plot for synthetic sample A illustrating average read depth per million reads for each targeted gene with GenCap-Seq enrichment. Read depths are plotted as blue lines around the circular PAO1 reference genome, and approximate genome coordinates are indicated at gray radial lines. Blue lines for four pairs of genes overlap due to close genome proximity (*algU/mucA*, *exsA/exsD*, *wspF/lasB*, and *ampD/pilD*). Concentric circles (light gray) indicate scale of average read depth per million reads sequenced.

Shotgun sequencing of single replicates from two synthetic samples (containing 2% P. aeruginosa DNA and 98% human DNA) without GenCap-Seq enrichment showed an average of 0.002% (range, 0.001 to 0.004%) total reads mapping to the targeted genes (Fig. 3A), providing an average read depth across those regions of 3.9 (range, 1.7 to 4.5) per million sequence reads. After gene-targeted GenCap-Seq of the same samples, an average of 78% of reads mapped to the targeted genes (an average of 5% of reads mapped to each gene; range, 1% to 11%; *P* < 0.0001 two-tailed paired *t* test compared to shotgun sequencing) (Fig. 3A), yielding an average read depth of 6,614 (range, 1,825 to 13,843) per million sequence reads (*P* < 0.0001 two-tailed paired *t* test compared to shotgun sequencing) (Fig. 3B; see also Fig. S1). We concluded that GenCap-Seq using amplicon-derived probes can highly enrich DNA from specific genes or genomic regions.

10.1128/mbio.01424-22.3 FIG S1 Representative circle plot for synthetic sample B, illustrating average read depth per million reads for each targeted gene with GenCap-Seq enrichment. Download FIG S1, DOCX file, 0.03 MB. Copyright © 2022 Hayden et al. 2022 Hayden et al. https://creativecommons.org/licenses/by/4.0/ This content is distributed under the terms of the Creative Commons Attribution 4.0 International license.

### GenCap-Seq enriches target DNA in complex clinical samples.

Enrichment of target DNA in clinical specimens could be challenging, as the nontarget DNA from other bacteria present could share homology with the intended target DNA and because hybridization efficacy could be compromised by interfering substances or target DNA degradation. Thus, we evaluated the outcome of whole-genome GenCap-Seq performed on two types of clinical samples that presented different potential obstacles.

**FIG 4:**
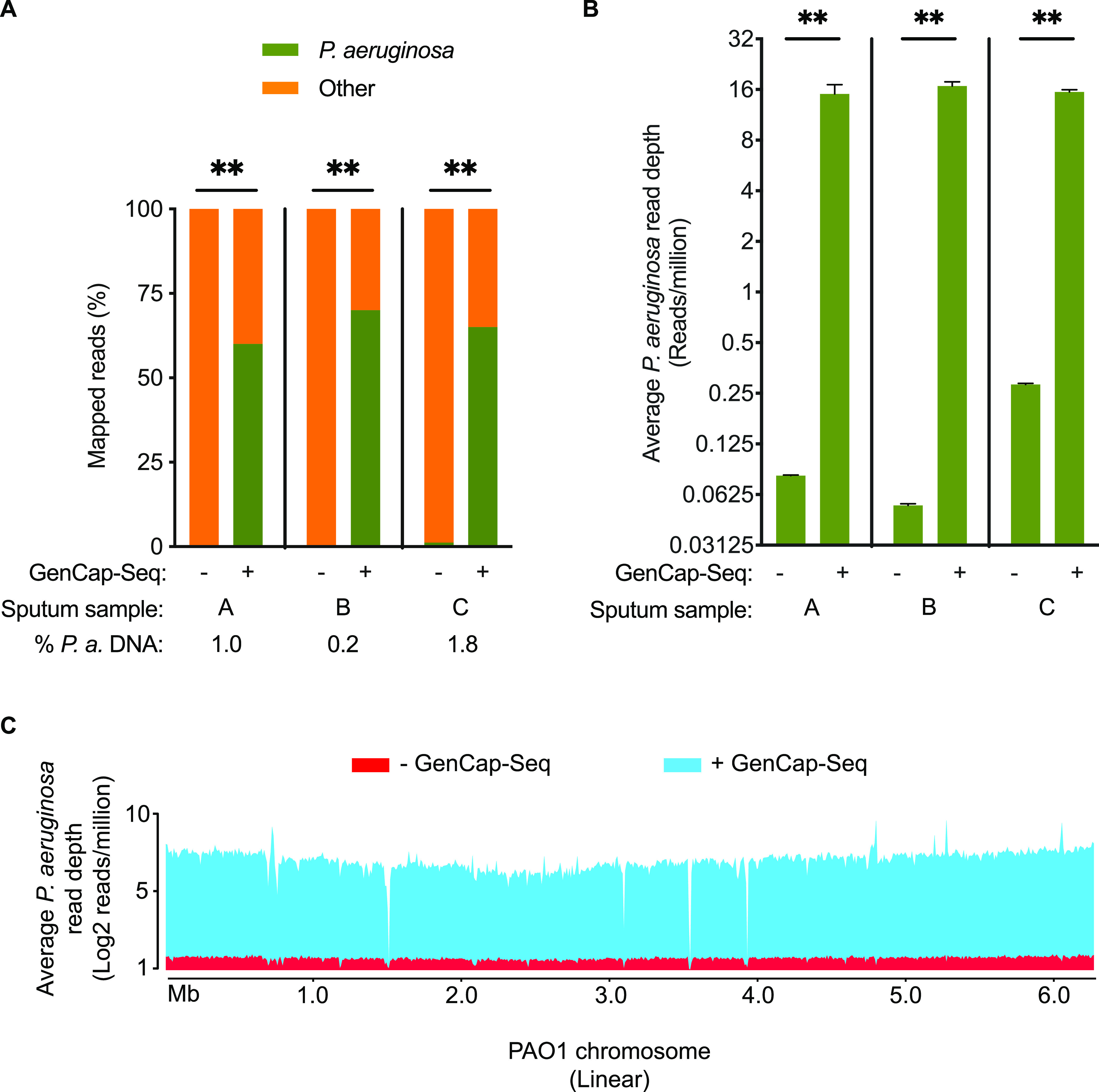
GenCap-Seq using whole-genome probes enriched P. aeruginosa DNA in CF patient sputum. (A) Percentages of sequence reads mapped to P. aeruginosa without (−) or with (+) GenCap-Seq enrichment in three clinical samples (samples A, B, and C). **, *P* < 0.01 by two-tailed paired *t* test. Stacked bars indicate mean percentages of sequence reads, from 2 (−) or 3 (+) technical replicates of each clinical sample, that align to P. aeruginosa (green) or human (orange). (B) Average read depth per million reads for P. aeruginosa without (−) or with (+) enrichment by GenCap-Seq of the replicates in panel A. **, *P* < 0.01 by two-tailed paired *t* test. (C) Average read depth per million reads sequenced that align to P. aeruginosa without (red) and with (blue) enrichment by GenCap-Seq across the 6.3-Mbp PAO1 reference genome. Data are integrated from all replicates examined in panel A.

We first tested CF sputum, which contains large quantities of human DNA (16), endogenous and pharmacologic nucleases and oxidants that could degrade DNA (17), and cationic molecules such as neutrophil elastase, myeloperoxidase, and histones that avidly bind negatively charged DNA (18). Quantitative PCR (qPCR) of sputum samples from three different people with CF had an average relative abundance of 1.0% P. aeruginosa DNA (range, 0.2% to 1.8%), with the remainder originating from host and other microbes. Consistent with this result, conventional shotgun sequencing yielded an average of 0.6% of reads mapping to P. aeruginosa (range, 0.3% to 1.2%) (Fig. 4A), providing an average read depth of 0.14 (range, 0.05 to 0.28) per million sequence reads (Fig. 4B). After GenCap-Seq using PAO1 probes, the proportion of reads mapping to P. aeruginosa increased to 65% (range, 60% to 70%, *P* < 0.01 two-tailed paired *t* test compared to shotgun sequencing) (Fig. 4A), yielding an average P. aeruginosa read depth of 16 (range, 13 to 18) per million sequence reads (*P* < 0.01 two-tailed paired *t* test, compared to shotgun sequencing) (Fig. 4B). As with synthetic samples, consistent enrichment was evident over the entire reference genome (Fig. 4C).

**FIG 5:**
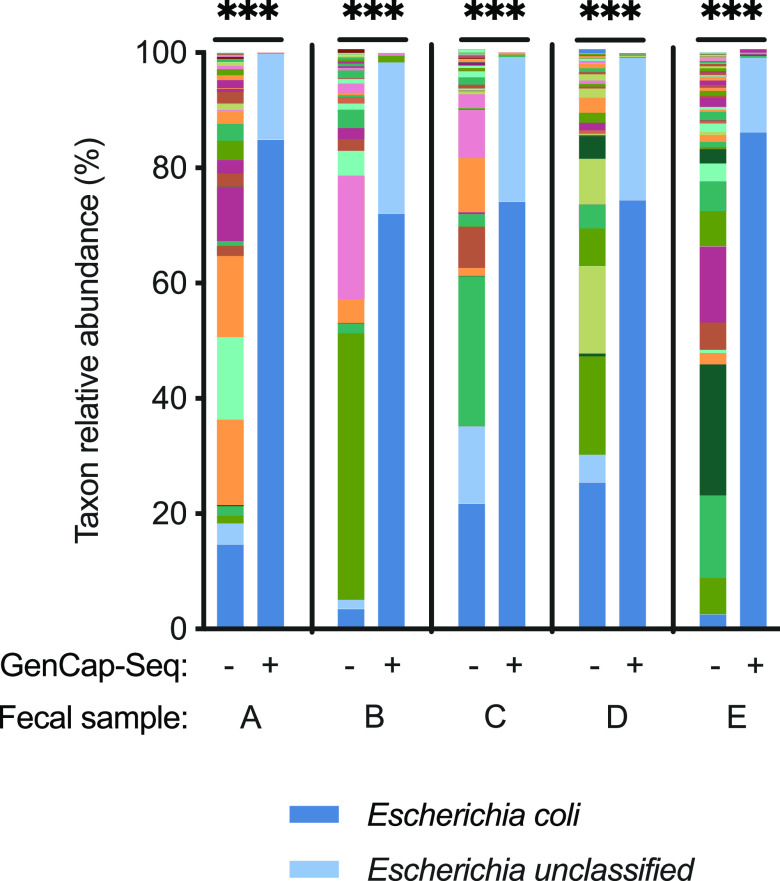
GenCap-Seq enriched E. coli DNA in fecal samples containing complex microbial communities. Results are shown for five clinical specimens (A through E). Stacked bars of various colors indicate percentages of relative abundance of different bacterial taxa without (−) and with (+) enrichment by GenCap-Seq using capture probes made from an E. coli isolate cultured from each specimen. ***, *P* < 0.001 by two-tailed paired *t* test. For clarity, the color codes for E. coli and Escherichia unclassified are displayed.

We separately tested GenCap-Seq on fecal samples, which contain little human DNA but large amounts of bacterial DNA from many species, including those that could be closely related to the targeted organism. We performed GenCap-Seq on fecal samples from 5 different individuals, and for each we used probes made from Escherichia coli isolates cultured from the sample itself. Metagenomic taxonomic analysis (19) was used to measure the extent and specificity of enrichment. The relative abundance of E. coli-derived reads from shotgun sequencing averaged 14% across the specimens (range, 2% to 25%) (Fig. 5). After GenCap-Seq, E. coli-derived reads increased in abundance to an average of 78% (range, 72% to 86%; *P* < 0.001 two-tailed paired *t* test compared to shotgun sequencing) (Fig. 5), with attendant decreases in other microbial taxa. Recovery of unclassified Escherichia species DNA was also proportionately increased, reflecting failure of the metagenomic classifier to identify some E. coli reads to the species level (see Table S1) and, potentially, cross-hybridization of E. coli probes to other Escherichia species. We concluded that GenCap-Seq enriches target DNA in clinical specimens in which target DNA is rare and potentially interfering templates and substances are abundant.

10.1128/mbio.01424-22.4 TABLE S1 MetaPhlAn reported relative abundance of E. coli and Escherichia from shotgun sequencing of fecal samples and individual E. coli isolates. Download Table S1, DOCX file, 0.1 MB. Copyright © 2022 Hayden et al. 2022 Hayden et al. https://creativecommons.org/licenses/by/4.0/ This content is distributed under the terms of the Creative Commons Attribution 4.0 International license.

### GenCap-Seq can enrich accessory genome content.

Bacterial genomes contain sequences that are shared across nearly all members of their species (the core genome) and those restricted to particular strains (the accessory genome) (20). In P. aeruginosa, accessory genes comprise on average ~10% of the genome (21) and can mediate pathogenesis functions, including motility, metabolism, virulence, and drug resistance (20). Thus, genetic variation in bacterial accessory genes and their changes over time could be important in disease.

We tested whether hybridization probes made from subjects’ own isolates could enrich strain-specific accessory genome content. Isolates from two CF subjects (designated A and B) were sequenced to generate draft genome assemblies for read mapping. For evaluation purposes, each nucleotide position in the assembly was considered core genome if present in the PAO1 reference, and accessory genome if absent from PAO1. Accessory content accounted for 10% and 6% of the isolate genomes of A and B, respectively.

**FIG 6:**
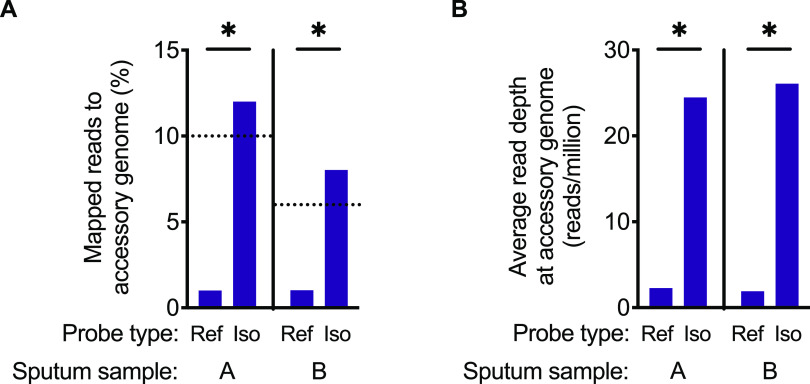
GenCap-Seq enriched the accessory genome of P. aeruginosa in CF patient sputum. GenCap-Seq using probes from the PAO1 reference strain (Ref) or isolate-specific (Iso) probes was carried out in two independent CF sputum samples, A and B. (A) Percentages of sequence reads mapped to accessory genome regions using PAO1 or isolate-specific probes. *, *P* < 0.05 by two-tailed unpaired *t* test. Dotted lines indicate percentages of accessory genome content measured for P. aeruginosa isolates from sample A (10%) and sample B (6%). (B) Average read depth per million reads sequenced for accessory genome regions using PAO1 or isolate-specific probes. *. *P* < 0.05 by two-tailed paired *t* test.

We performed GenCap-Seq on sputum samples from these subjects using whole-genome probes generated either from subject-derived clinical isolates or from the PAO1 reference strain. GenCap-Seq using PAO1 probes resulted in an average of 0.9% reads (0.7% reads for A, 1.3% reads for B) mapping to accessory positions (Fig. 6A), yielding an average read depth at accessory positions of 2.1 per million sequence reads (1.9 for A, 2.3 for B) (Fig. 6B). In contrast, GenCap-Seq using probes made from the subjects’ own isolates resulted in 12% and 8% reads mapping to accessory regions in A and B, respectively (*P* < 0.05 two-tailed unpaired *t* test compared to PAO1 probes), approximating the measured accessory genome content of those strains (Fig. 6A). Additionally, isolate-specific probes yielded average read depths of 24 and 26 per million sequence reads for A and B, respectively (*P* < 0.05 two-tailed paired *t* test compared to PAO1 probes) at accessory genome positions (Fig. 6B). Notably, the ratio of accessory to core sequence read depth averaged 0.1 for GenCap-Seq with PAO1 probes and 1.0 after GenCap-Seq with isolate probes, indicating that use of isolate-matched probes resulted in approximately equal read coverage across core and accessory genome content.

### GenCap-Seq accurately reports nucleotide variant frequencies in synthetic mixtures.

In addition to enriching for low-abundance bacterial DNA, GenCap-Seq’s performance depends on the accuracy of variant frequency calls. Several factors could complicate variant frequency measurements, including secondary DNA structure, sequence differences between probe and sample DNA, and PCR bias during library preparation and post-hybridization amplification.

**FIG 7:**
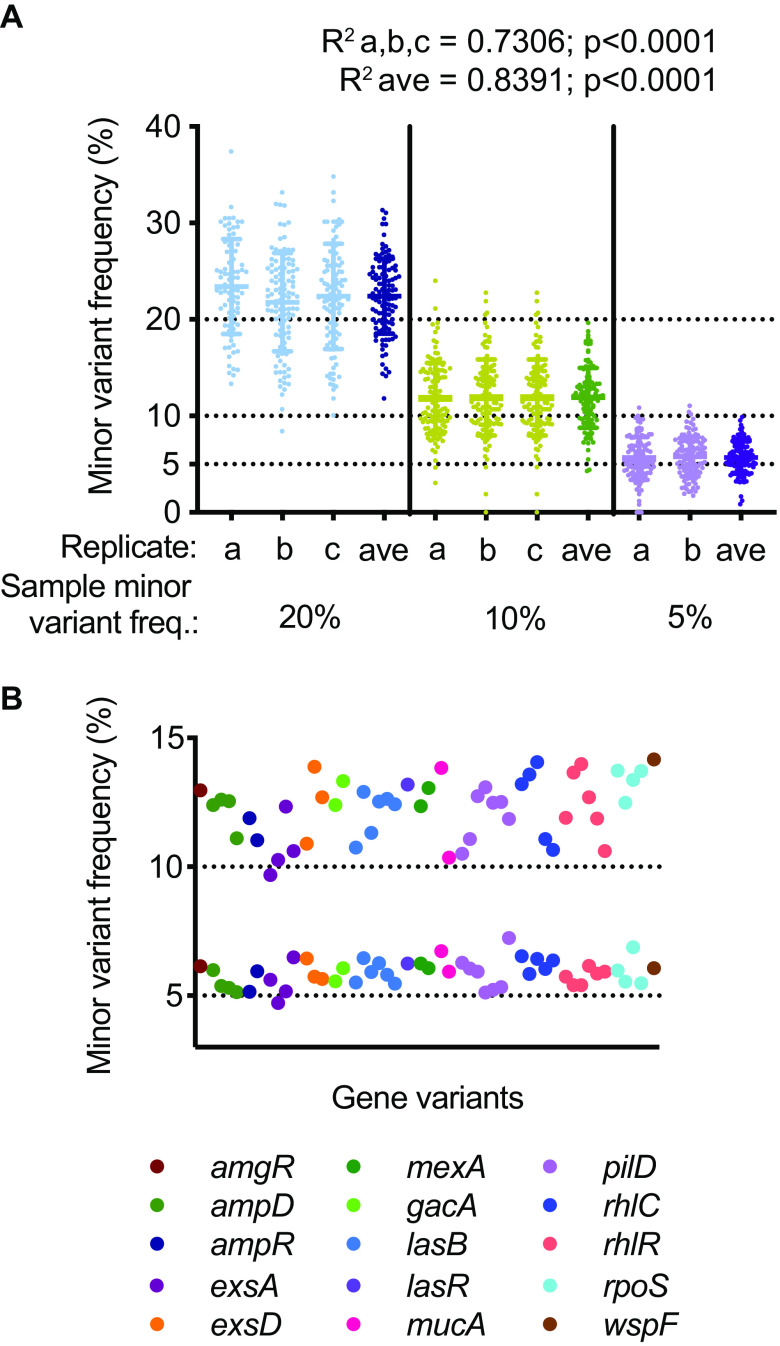
GenCap-Seq accurately determined nucleotide variant frequencies in synthetic samples. (A) Observed minor variant frequencies for 113 genome positions are shown for technical replicates (a, b, and c) and the average of replicates (ave) for three synthetic samples containing minor variant frequencies of 20%, 10%, and 5% (DNA from strains PAO1 and PACS2 combined in ratios of 80:20, 90:10, and 95:05, respectively). Solid horizontal lines indicate means and standard deviations. Expected minor variant frequency lines (dotted) are indicated. Linear regression of observed median minor variant frequencies correlated with the expected (*R*^2^ a,b,c = 0.7306, average *R*^2^ = 0.8391). (B) Minor variant frequencies for 50 positions in 15 genes for two synthetic samples with minor variant frequencies of 10% and 5% (PAO1 to PACS2 DNA at 90:10 and 95:05, respectively). Expected minor variant frequency lines (dotted) are indicated. Variants are color-coded by gene.

We first tested the accuracy of variant frequency quantitation by whole-genome GenCap-Seq using the synthetic mixtures described above, which combined PAO1 and PACS2 strains at 3 proportions (80:20, 90:10, and 95:05) in a background of 98% human DNA. We measured minor variant frequencies at 113 positions present in 20 core genes distributed throughout the P. aeruginosa genome, where single-nucleotide polymorphisms distinguished the two strains (Fig. 7A; see also Data Set S1, Sheet 1). The observed minor variant frequencies were highly correlated with expected values at each proportion tested (*R*^2^ = 0.7306) (Fig. 7A). The coefficient of variation for individual measurements (see Table S2) was inversely proportional to the minor variant frequency, likely reflecting the effects of Poisson sampling. Integrating multiple independent measurements from a single specimen could potentially improve the accuracy of variant measurements, so we also assessed variant quantitation when averaged across multiple technical replicates (Fig. 7A). We found that averaging replicate measurements improved correlation with expected values (*R*^2^ = 0.8391) and reduced the coefficient of variation (see Table S3).

10.1128/mbio.01424-22.5 TABLE S2 Coefficient of variation for individual measurements of 113 positions present in 20 core genes where single nucleotide polymorphisms distinguished the PAO1 and PACS2 P. aeruginosa strains. Download Table S2, DOCX file, 0.02 MB. Copyright © 2022 Hayden et al. 2022 Hayden et al. https://creativecommons.org/licenses/by/4.0/ This content is distributed under the terms of the Creative Commons Attribution 4.0 International license.

10.1128/mbio.01424-22.6 TABLE S3 Coefficient of variation for measurements averaged across replicates of 113 positions present in 20 core genes where single nucleotide polymorphisms distinguished the PAO1 and PACS2 P. aeruginosa strains. Download Table S3, DOCX file, 0.02 MB. Copyright © 2022 Hayden et al. 2022 Hayden et al. https://creativecommons.org/licenses/by/4.0/ This content is distributed under the terms of the Creative Commons Attribution 4.0 International license.

10.1128/mbio.01424-22.11 DATA SET S1 Minor allele frequencies of 113 genome positions from 20 core genes distributed throughout the P. aeruginosa genome (Sheet 1) and significant intersections of genes that lost diversity during tobramycin treatment among ≥3 of 5 subjects (Sheet 2). Download Data Set S1, XLSX file, 0.04 MB. Copyright © 2022 Hayden et al. 2022 Hayden et al. https://creativecommons.org/licenses/by/4.0/ This content is distributed under the terms of the Creative Commons Attribution 4.0 International license.

We separately tested the accuracy of variant frequency calls for gene-targeted GenCap-Seq performed using amplicon-derived probes. Fifteen of the 17 genes selected for targeted enrichment experiments harbored one to seven single-nucleotide polymorphisms each that distinguished PAO1 from PACS2, representing a total of 50 individual sites. Across these regions, average minor variant frequencies measured 12% and 6% for the 90:10 and 95:05 mixtures, respectively (Fig. 7B; see also Table S4), in accordance with expectations. Taken together, these results indicated high agreement between expected and measured variant frequencies by GenCap-Seq.

10.1128/mbio.01424-22.7 TABLE S4 Minor variant frequencies for 50 variable positions across 15 core genes for the 90:10 and 95:05 synthetic samples. Download Table S4, DOCX file, 0.04 MB. Copyright © 2022 Hayden et al. 2022 Hayden et al. https://creativecommons.org/licenses/by/4.0/ This content is distributed under the terms of the Creative Commons Attribution 4.0 International license.

### GenCap-Seq accurately determines variant frequencies in sputum.

Testing GenCap-Seq’s accuracy for variant frequency measurements in human samples required an independent benchmark against which to compare results. To generate this, we processed freshly expectorated CF sputum samples using a procedure that depletes human DNA by selectively lysing host cells and enzymatically digesting extracellular DNA before bacterial lysis (22). This approach requires freshly collected samples to preserve the integrity of host cells, considerable sample volumes, and high-depth sequencing, making it challenging for routine use.

**FIG 8:**
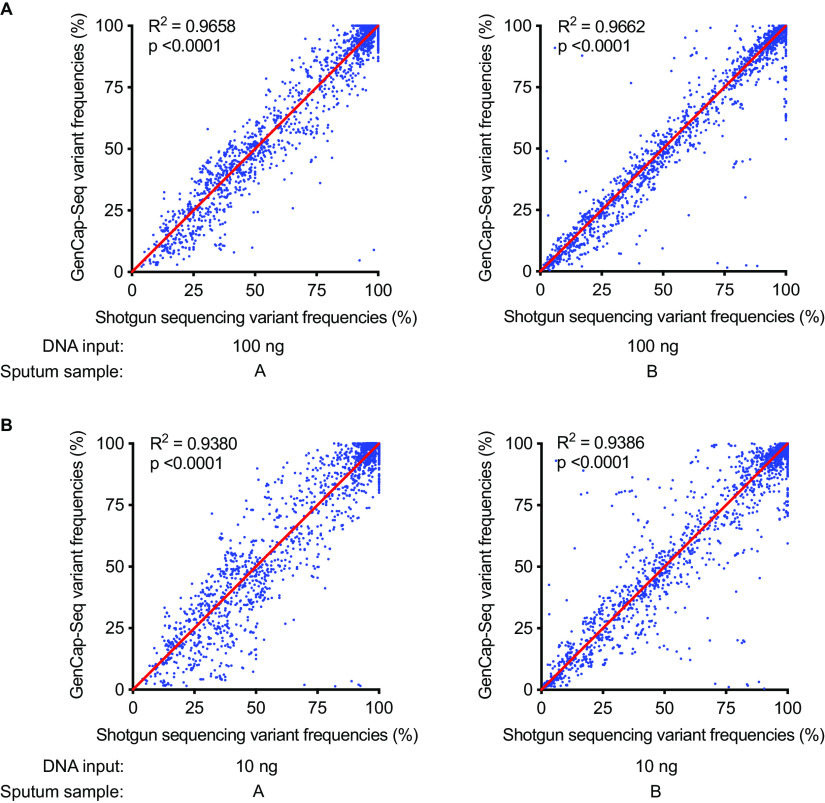
GenCap-Seq accurately determined nucleotide variant frequencies in clinical samples. (A) Linear regression plot of variant frequencies for two independent CF sputum samples (A and B) with standard DNA input (100 ng) determined following enrichment with GenCap-Seq using PAO1 probes and directly using shotgun metagenomic sequencing (*R*^2^ > 0.96). (B) Linear regression plot of variant frequencies for the same sputum samples with low DNA input (10 ng) determined following enrichment with GenCap-Seq using PAO1 probes and directly using shotgun metagenomic sequencing (*R*^2^ > 0.93). Each dot indicates the frequency of a variant position as determined by read alignment to the PAO1 reference genome.

We collected sputum from two P. aeruginosa-infected CF subjects, each containing P. aeruginosa DNA at ~1% relative abundance as measured by qPCR. We processed a fresh aliquot of each sputum using the differential lysis procedure, then subjected the resultant DNA to shotgun sequencing. A second aliquot from each sample was frozen for 2 weeks at −80°C before undergoing GenCap-Seq using whole-genome probes made from PAO1. Variants were called relative to the PAO1 reference genome. GenCap-Seq detected 99% of the variant positions identified by shotgun sequencing in each of the two samples (28,118 of 28,297 and 28,732 of 28,922 sites), and linear regression indicated a strong correlation between variant frequencies detected by shotgun sequencing and GenCap-Seq (*R*^2^ > 0.96) (Fig. 8A).

Clinical specimens may be limited by volume and DNA concentrations, so we separately assessed GenCap-Seq’s ability to measure variant frequencies in frozen sputum aliquots using 10 ng instead of the standard 100 ng of input DNA. GenCap-Seq detected 98% of the variant positions identified by shotgun sequencing in each of the two low-input samples (27,770 of 28,297 and 28,274 of 28,922 sites). Moreover, variant frequency measurements from the shotgun and low-DNA input GenCap-Seq data sets remained highly correlated (*R*^2^ > 0.93) (Fig. 8B).

Together, these results showed that GenCap-Seq accurately determines variant frequencies of bacterial strains in clinical specimens containing a vast excess of nontarget DNA and DNA-binding and degrading substances, even when small amounts of sample DNA are available.

### GenCap-Seq reveals reduced P. aeruginosa diversity after tobramycin therapy.

We used GenCap-Seq in proof-of-principle studies investigating the responses of diversified clonal P. aeruginosa CF infections to antibiotic treatment *in vivo*. Intrastrain diversity could contribute to poor treatment outcomes if variants that resist antibiotic killing *in vivo* help maintain infecting biomass when sensitive variants are cleared. If such a mechanism were operative, intrastrain diversity would be expected to decline during treatment. However, it is alternatively possible that evolved diversity is immaterial to treatment responses under *in vivo* conditions. For example, slow bacterial growth, bacterial aggregation, or drug inactivation could produce physiological tolerance phenotypes that are nonspecific and independent of intrastrain genetic variation.

To investigate these possibilities, we utilized GenCap-Seq data to calculate π (23), a metric of nucleotide diversity within populations, in CF patient sputum obtained before and after antibiotic therapy. Briefly, π measures the number of pairwise nucleotide differences at every genomic position in all corresponding sequences (in this case, sequence reads covering the same base) generated from the sample.

We performed GenCap-Seq using probes made from PAO1 DNA on seven CF subjects chronically infected with P. aeruginosa who were prescribed inhaled tobramycin cycled in monthly periods of “On” and “Off” treatment (24). All subjects were infected by a single P. aeruginosa strain, as determined by population-level multilocus sequence typing (25) (see Table S5). We used the resultant variant frequency measurements to calculate genome-wide π during the tobramycin Off periods and 1 to 2 weeks into the On periods of tobramycin treatment, when P. aeruginosa sputum culture density was near its nadir (24). Samples were stored frozen before analysis, and for most subjects only limited quantities of DNA were available.

10.1128/mbio.01424-22.8 TABLE S5 Results of population MLST for 7 CF subjects before tobramycin treatment. Download Table S5, DOCX file, 0.04 MB. Copyright © 2022 Hayden et al. 2022 Hayden et al. https://creativecommons.org/licenses/by/4.0/ This content is distributed under the terms of the Creative Commons Attribution 4.0 International license.

**FIG 9:**
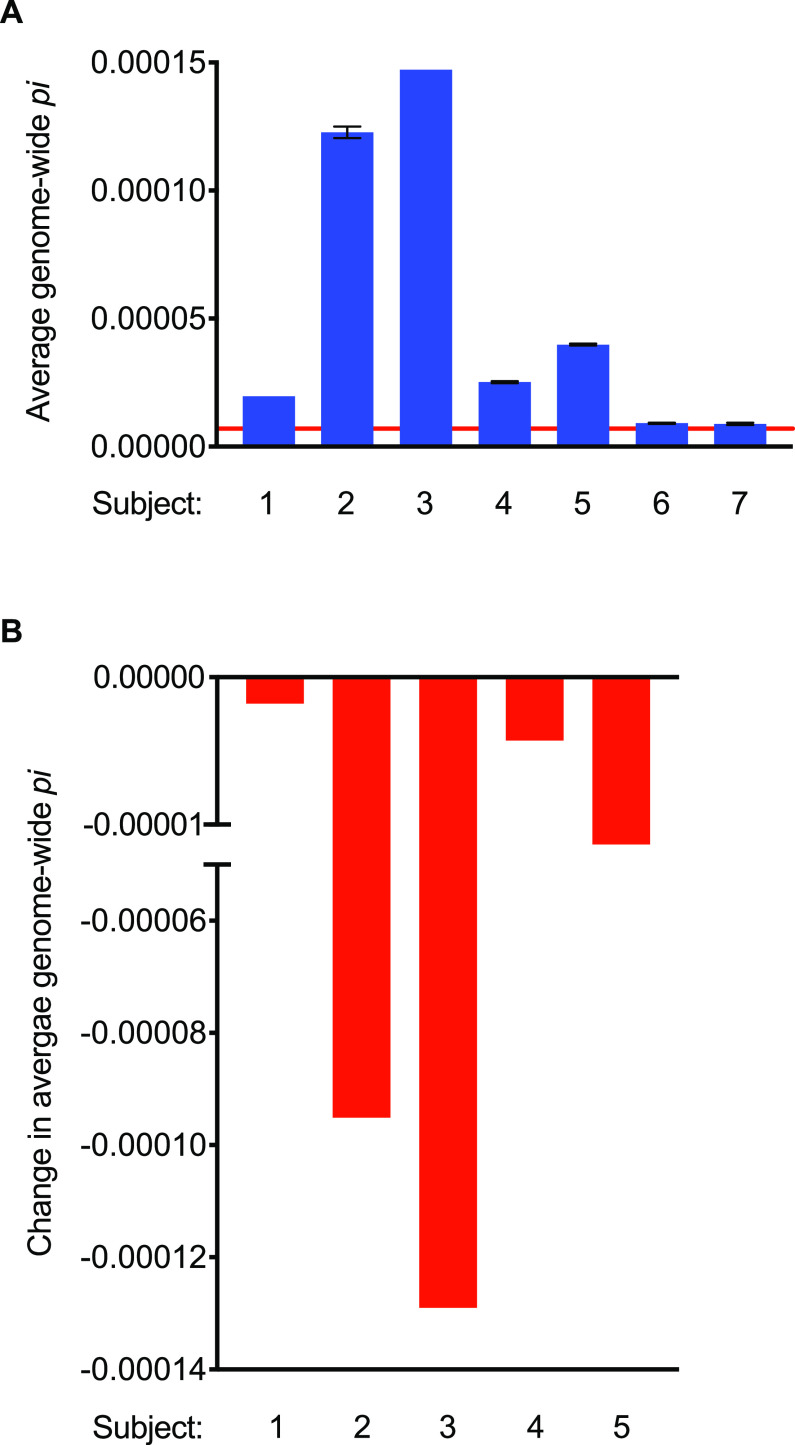
Genetic diversity of P. aeruginosa populations decreased with tobramycin therapy. (A) Average genome-wide π values for Off treatment are shown for seven subjects (1 through 7). The red solid line at 7.0 × 10^−6^ indicates the average genome-wide π value for self-mapping of simulated reads for the PAO1 reference genome and is thereby considered the limit of detection. Subjects 6 and 7 are at the limit of detection. (B) Change in average genome-wide π values following tobramycin therapy for the 5 subjects with initially measurable π values.

In two of the seven subjects studied (subjects 6 and 7), genome-wide π was at the lower limit of detection during the Off treatment period (Fig. 9A), being indistinguishable from error-free sequence reads simulated from the PAO1 reference genome itself (π = 7.0 × 10^−6^). The absence of measurable P. aeruginosa genetic diversity in these individuals could reflect insufficient time for the infecting population to diversify (data on infection duration were unfortunately not available), the strains’ inherent capacities to diversify, or other subject-specific factors. In the remaining 5 subjects, π values during Off treatment periods were greater than the limit of detection and in all cases decreased during treatment (Fig. 9B; see also Table S6). Subjects 2 and 3 showed the highest Off tobramycin π values and also the largest reductions in π during therapy. Although our study examined a small number of samples and lacked formal selection analysis, these results raised the possibility that the diversified isolates present in an individual before treatment differed materially in fitness during *in vivo* tobramycin exposure and that tobramycin may cause purifying selection in genetically diverse P. aeruginosa populations.

10.1128/mbio.01424-22.9 TABLE S6 Average genome-wide π, change in average genome-wide π from “off” to “on” tobramycin, and number of core genes and genes with nonzero π values for subject samples. Download Table S6, DOCX file, 0.03 MB. Copyright © 2022 Hayden et al. 2022 Hayden et al. https://creativecommons.org/licenses/by/4.0/ This content is distributed under the terms of the Creative Commons Attribution 4.0 International license.

### Tobramycin therapy reduces allelic diversity in genes without traditional resistance functions.

We next used GenCap-Seq to identify individual genes that exhibited allelic diversity during antibiotic Off periods but no diversity during antibiotic On periods. Such treatment-induced reductions in gene-level diversity would suggest that allelic variation in the genes is tolerated in the absence of antibiotic, but not in its presence. Consequently, genes exhibiting this pattern may be critical for bacterial fitness during *in vivo* antibiotic exposure. An important caveat is that some genes will exhibit artifactual reductions in diversity due to linkage with others that are the actual targets of antibiotic selection. However, genes that repeatedly show treatment-induced diversity reductions across different subjects are likely to be under selection themselves, as strain-specific variation in genomic structure results in different alleles being linked in unrelated strains (26).

The majority of core genes exhibited no measurable diversity (π = 0) in subjects’ sputum, regardless of antibiotic treatment status. However, in the 5 subjects with measurable genome-wide π values, an average of 958 genes per subject (range, 107 to 1,845) had π values greater than zero at the Off treatment time point and an average 816 (range, 75 to 1,727) genes per subject lost all diversity (i.e., π = 0) during On treatment (see Table S6). Subject 3 demonstrated the highest proportion of genes where allelic diversity was eliminated during tobramycin treatment (97% or 1,727/1,782 genes), whereas subject 4 had the lowest proportion (38% or 198/515 genes).

We searched for significant intersections (*P* < 0.05 by SuperExactTest) (27) of genes that lost diversity during treatment among 3 or more of the 5 subjects. We found 205 genes common to 3 subjects and 4 genes common to 4 subjects (see Data Set S1, Sheet 2). In all cases, the major allele present during the tobramycin Off period went to fixation during treatment. This may be related to the fact that all studied subjects used regularly cycled tobramycin, as chronic drug exposure can promote compensatory mutations that reduce the fitness costs of resistance alleles enabling them to predominate even when drug is absent (28).

We examined the functional annotation of genes in which diversity was lost across multiple subjects, and we identified several trends. One category of genes is known to increase resistance to aminoglycosides (*fusA1*, *mexA*, *oprM*, *parE*, *pchF*, PA4292) or other antibiotics (*ccmE*, *gyrB*, *mexD*, *mexF*) under *in vitro* conditions (29). Others mediate functions involved in general stress tolerance, including bacterial aggregation (*wspA*, *wspF*, *narX*) (30, 31), and membrane construction and transport (*wbpM*, *wzt*, *dacC*, *pbpG*, *ureC*, *glmS*) (32–36). We also found diversity loss in some metabolic genes (*gltB*, *gltD*, *purH*, *lysA*, *sucA*), reported to affect resistance perhaps by slowing growth or affecting drug target activity (37). Although this analysis remains exploratory due to our limited sample size, these data illustrate how GenCap-Seq data could help detect changes in diversified bacterial populations during important clinical events and identify the specific bacterial genes and functions that mediate bacterial survival during treatment.

## DISCUSSION

The lack of facile measurement methods has made it difficult to study the consequences of intrastrain diversity on disease. Here, we have shown that GenCap-Seq can accurately measure intrastrain genetic variation in specimens that are heavily contaminated by human and nontarget bacterial DNA and contain abundant host-derived DNA-binding and -degrading substances.

Several methods have previously been developed to reduce the impact of nontarget DNA on bacterial genomic analysis, but complications inherent to clinical specimens limit their use. One strategy selectively reduces host DNA, either by differential cell lysis and DNase treatment or by antibody depletion (38–40), but can be overwhelmed if eukaryotic DNA is in vast excess. In addition, clinical specimens often must be frozen prior to analysis, and bacterial lysis during thawing can compromise selective digestion by making target DNA accessible to extracellular DNases. Furthermore, lysis and antibody-mediated approaches do not reduce nontarget bacterial DNA, and samples like feces and saliva contain hundreds of bacterial taxa at high densities. Other approaches rely on hybridization of target bacterial DNA to actively enrich targets of interest (41–43) but utilize costly commercially synthesized probes and would be both challenging and cost prohibitive to implement on a genomic scale. Earlier methods that bypass commercial probe synthesis by generating hybridization probes from PCR products (44) would scale poorly to enriching entire bacterial genomes and would require optimization for individual taxa. Alternatively, methods that directly sequence PCR-amplified target DNA (15) are more cost-effective but can introduce bias during amplification and restrict allele frequency measurements to predefined subsets of the genome.

GenCap-Seq provides several advantages over existing approaches for selectively sequencing target DNA. First, because users inexpensively generate hybridization probes using DNA templates of their choosing, they control the content that gets enriched and depleted and can interrogate diversity and allele frequency measurements from individual genes to the entire genome of targeted species, including strain-specific accessory genes. Findings in the accessory genome could be particularly novel, as accessory genes encode important pathogenesis functions (20). Second, GenCap-Seq is compatible with archival samples that have been frozen, are of limited quantity, and contain minute amounts of the organisms of interest. Third, since the DNA fragments that are ultimately sequenced have unique start and end sites due to random DNA fragmentation during library preparation, the number of individual bacterial chromosomes, and attendant polymorphisms, interrogated at each nucleotide position can be accurately estimated.

GenCap-Seq also has limitations. Because hybridization is used, conserved genetic elements (including portions of 16S rRNA) can be unintentionally enriched from nontargeted species, and these could produce error. The DNA fragmentation required during sequencing library preparation makes it impossible to directly establish linkage between measured variants unless they are close enough to be represented in an individual sequence read. Despite its capacity for efficient enrichment, GenCap-Seq is likely to be challenging in specimens where bacterial biomass is extremely low or in those for which robust sequencing libraries are difficult to prepare, as these cases may not result in optimal hybridization. Finally, while the data presented here showed GenCap-Seq functions well when used on two complex clinical samples (sputum and feces), it is possible that factors specific to other sample types could interfere with the method.

Our pilot work studying CF subjects undergoing cyclic inhaled tobramycin treatment illustrates how GenCap-Seq could increase understanding of infection biology. The reduction in intrastrain P. aeruginosa diversity we observed is consistent with an ecological concept termed the “insurance effect,” wherein the relative abundance levels of subpopulations change as selective conditions vary (8). Population composition changes provide a form of biological insurance, because subpopulations with different fitness profiles sustain population biomass at different times. Most work demonstrating insurance effects involve species-level diversity in ecological settings, yet our findings raised the possibility that intrastrain bacterial diversity evolving during chronic infection has similar effects. Given the extensive functional diversity found in evolved strains (1), variant abundance changes could contribute to the ability of pathogens causing chronic infections to resist many stresses during chronic infection, including immune responses, antibiotics, and nutritional limitations.

Our analysis of diversity changes occurring by gene was also informative, as it produced a candidate list of genes losing diversity during treatment that could be critical for maintaining bacterial fitness during *in vivo* antibiotic exposure (see Data Set S1, Sheet 2). Some genes found to repeatedly lose diversity are known to have direct roles in aminoglycoside resistance under *in vitro* conditions. Others could affect tobramycin resistance in more complicated and environmentally influenced ways, such as by mediating bacterial aggregation, modulating membrane integrity, or affecting nutritional and metabolic functions that operate *in vivo*.

Larger confirmatory studies are needed, as the number of genes exhibiting loss of diversity due to linkage will decrease as more subjects are examined. However, these findings raise the possibility that previously unappreciated mechanisms may mediate bacterial fitness during *in vivo* tobramycin exposure, including some that would not be apparent or identified via *in vitro* studies. For example, our analysis registered only 8 of the 117 P. aeruginosa genes identified as being negatively selected by tobramycin exposure in an *in vitro* transposon mutagenesis study (45) (*chpA*, *gltP*, *oprM*, *pstC*, *trkA*, PA5133, PA0667, and PA5471), but it implicated an additional 198 genes not found by that work. While several mechanisms could explain minimal overlap between the data sets (e.g., lab strain versus clinical isolates or selection of transposon mutants versus allelic variants), differences in genes’ resistance effects or fitness costs of mutation *in vitro* and *in vivo* may be a key factor (46).

While the evolved diversity of infecting populations could pose major challenges to treatment, methods like GenCap-Seq that enable genome-wide measurement of allele frequency in infecting populations could improve understanding of chronic infections and foster new treatment approaches. One question for future research is whether the extent of intrastrain diversity affects treatment responses or infection severity. This information could have prognostic value, help optimize treatment timing, and focus attention on approaches to limit diversification of infecting strains. Genome-wide allele frequency measurements could also enable tests of approaches that serially apply “purifying” stresses to successively reduce infecting population diversity. In addition, if the abundance of variants in particular genes or pathways were found associated with disease flares or progression, it might be possible to manipulate population composition toward a less injurious state by exploiting fitness costs of particular variants. GenCap-Seq could provide advantages in other applications where isolating specific organisms or sequences from complex specimens would be beneficial, including the sensitive diagnosis of infectious disease, or potentially, the depletion of host DNA for metagenomic studies.

## MATERIALS AND METHODS

### Clinical samples.

Sputum samples used in P. aeruginosa enrichment and variant frequency experiments were obtained from cystic fibrosis patients with chronic P. aeruginosa infections who attended clinic visits at the University of Washington Medical Center. Fecal samples used in E. coli enrichment experiments have been previously described (24, 47).

Sputum samples used in our proof-of-principle study of P. aeruginosa diversity before and during tobramycin therapy were collected from patients with CF as part of the Tobramycin Inhaled Powder (TIP) study (24). Briefly, spontaneously expectorated sputum was collected prospectively from subjects prior to and weekly during the month-long maintenance treatment period, which occurred after at least 28 days without exposure to any antibiotics other than maintenance azithromycin.

### Ethics statement.

Samples were collected from patients in accordance with the University of Washington Institutional Review Board (approved protocol number 06-4469). Patients provided written informed consent prior to collection of samples.

### DNA extraction.

For synthetic samples, genomic DNA was extracted from overnight cultures of P. aeruginosa strains PAO1 and PACS2, and human DNA was extracted from HeLa cells, using the DNeasy blood and tissue kit (Qiagen). P. aeruginosa and human DNAs were quantified using the Qubit double-stranded (dsDNA) broad range assay kit (Thermo Fisher Scientific) prior to their combination.

All sputum samples were diluted 1:1 in 0.1% Sputolysin (MilliporeSigma) for homogenization prior to DNA extraction. DNA was extracted from 100-μL aliquots of previously frozen sputum samples using the DNeasy PowerSoil kit (Qiagen) following the manufacturer’s protocol. DNA was extracted from 100-μL fresh sputum samples within 24 h of expectoration by using the QIAamp DNA Microbiome kit (Qiagen), which allows for selective lysis of host DNA, using 10 mM Tris-Cl (pH 8.5) for DNA elution. DNA extraction from fecal samples was as described previously (47).

Genomic DNA from relevant P. aeruginosa and E. coli strains was extracted for probe preparation using the DNeasy blood and tissue kit (Qiagen).

### Probe preparation.

The step-by-step protocol for whole-genome probe preparation is provided in the supplemental material (see Text S1). Briefly, template DNA (genomic or amplicon) was quantified using the Qubit dsDNA broad-range assay kit (Thermo Fisher Scientific) and sheared using a M220 focused ultrasonicator (Covaris) to ~150-bp fragments. Sheared DNA was repaired using the NEBNext FFPE DNA repair mix (New England BioLabs) and then purified and size selected using Ampure XP beads (Beckman Coulter Life Sciences). DNA fragments were dephosphorylated with shrimp alkaline phosphatase (New England BioLabs) and biotinylated using terminal transferase (New England BioLabs) and biotin-11-dideoxy ATP (PerkinElmer). Biotinylated DNA was purified using a Monarch PCR and DNA cleanup kit (New England BioLabs) following the manufacturer’s protocol. DNA fragments were bound to Dynabeads MyOne streptavidin C1 (Thermo Fisher Scientific) followed by denaturation with NaOH to eliminate nonbiotinylated strands. Biotinylated single-stranded DNA (ssDNA) was dissociated from beads using formamide and was cleaned again using the Monarch PCR and DNA cleanup kit according to the manufacturer’s modified ssDNA protocol. DNA was eluted in 10 mM Tris HCl (pH 8.5) and stored at −20°C until further use.

Targeted GenCap-Seq included 17 core P. aeruginosa genes located throughout the PAO1 reference genome that have been associated with pathogenicity in CF chronic infections: *algU* (PA0762), *amgR* (PA5200), *ampD* (PA4522), *ampR* (PA4109), *exsA* (PA1713), *exsD* (PA1714), *gacA* (PA2586), *lasB* (PA3724), *lasR* (PA1430), *mexA* (PA0425), *mexD* (PA4598), *mucA* (PA0763), *pilD* (PA4528), *rhlC* (PA1130), *rhlR* (PA3477), *rpoS* (PA3622), and *wspF* (PA3703). Primer sets used to amplify genes for targeted GenCap-Seq probe construction are listed in Table S7.

10.1128/mbio.01424-22.10 TABLE S7 Primers used to amplify genes for targeted GenCap-Seq probe construction. Download Table S7, DOCX file, 0.1 MB. Copyright © 2022 Hayden et al. 2022 Hayden et al. https://creativecommons.org/licenses/by/4.0/ This content is distributed under the terms of the Creative Commons Attribution 4.0 International license.

### Library preparation, target capture, and sequencing.

DNA sequencing libraries were constructed as described previously (48), except for low-input (10-ng template) and proof-of-principle study sequencing libraries, which were constructed using DNA Prep with Enrichment Tagmentation reagents (Illumina). Libraries were quantified using the Qubit dsDNA high-sensitivity kit (Thermo Fisher Scientific) and enriched using the appropriate capture probe set (see Text S1 for the detailed probe hybridization and target DNA enrichment protocol). Briefly, probes were allowed to hybridize to target library DNA fragments, and bound fragments were purified with magnetic streptavidin beads using the xGen hybridization and wash kit (Integrated DNA Technologies). Enriched libraries were amplified using 10 to 12 PCR cycles and were sequenced on the NextSeq500 (Illumina) using 300-cycle paired-end sequencing.

### Sequence read processing, mapping, and coverage.

Raw sequence reads were trimmed for quality (phred score *Q* of <20), and Illumina adaptor and index sequences were removed, using Trimmomatic v0.33 (49). Duplicate reads were removed using EstimateLibraryComplexity, which is part of the Picard Tools package (https://broadinstitute.github.io/picard/), and the Sequniq v0.1 Python package (https://github.com/standage/sequniq). All software packages were run using default settings. For percent read mapping and genome coverage analyses, trimmed and deduplicated reads were aligned to the relevant P. aeruginosa genome (i.e., PAO1 or subject-specific isolate) or to the human genome (hg19) using Minimap2 v2.22 (50). Alignment coverage was enumerated using PySAM v0.16.0.1 (https://github.com/pysam-developers/pysam).

### Variant analysis.

Sequence reads were aligned to the appropriate reference genome using Minimap2 v2.22 (50), and variant frequencies were determined using Lofreq* (51). A minimum of 200× sequence read coverage was required in order to include a variant position in frequency analyses.

### Quantitative PCR.

Total bacterial and P. aeruginosa loads were determined by universal 16S and *gyrB* qPCR, respectively, with iTaq universal probe supermix (Bio-Rad) using primers and reaction conditions as described previously (52). Samples were analyzed on the CFX96 Touch real-time PCR detection system (Bio-Rad) with CFX Manager v3.1 software. Bacterial quantification was determined using standard curves of P. aeruginosa PAO1 DNA standard and TaqMan control genomic DNA (human) (Thermo Fisher Scientific). To determine the relative abundance of P. aeruginosa in clinical samples, the corresponding concentration of PAO1 DNA from a standard curve was divided by the total sample DNA concentration quantified using the Qubit dsDNA broad range assay kit (Thermo Fisher Scientific).

### Metagenomic taxonomic analysis of fecal samples.

MetaPhlAn2 v2.6.0 (19) was used with default settings to profile microbial community composition from metagenomic shotgun sequencing of fecal samples. Raw sequence reads were quality filtered as described above, and human DNA sequence was identified and removed using KneadData v0.7.2 (http://huttenhower.sph.harvard.edu/kneaddata) with the hg19 human reference genome. Duplicate reads were removed using hts_SuperDeduper as implemented through htstream-1.0 (https://github.com/s4hts/HTStream). Species with maximum abundance of <0.01% across all samples were removed to minimize sampling noise, and taxonomic proportions were rescaled to reflect the relative abundance of remaining taxa.

### Statistical analysis of bacterial DNA enrichment and nucleotide variant reporting.

All statistical analyses for DNA enrichment and nucleotide variant frequency experiments were conducted in Prism 9.0.2 (GraphPad). Two-tailed *t* tests were performed between various groups as described in Results and figure legends. Correlation of observed and expected minor variant frequencies in synthetic mixes, as well as of variant frequencies detected by shotgun sequencing and GenCap-Seq in sputum, was determined using simple linear regression.

### Nucleotide diversity of P. aeruginosa populations.

Nucleotide diversity was estimated using π, which measures the number of pairwise nucleotide differences at every genomic position in all corresponding sequences (23). The P. aeruginosa PAO1 reference sequence was first masked using RepeatMasker v4.1.0 (https://www.repeatmasker.org), and custom code (https://github.com/marade/PiDiv) was used to extend masked regions. Simulated reads of PAO1 were generated with DWGSIM v0.1.13 (https://github.com/nh13/DWGSIM) to determine the lower limit of detection for π.

Sequence reads for each subject were randomly subsampled (*n* = 10) to a uniform 100× coverage, except where noted in Table S6, in order to limit the effects of differential coverage across samples. Average genome-wide π values were calculated from all coding nucleotide positions with a minimum of 5× sequence read coverage. The π values for individual genes were calculated as the average across nucleotide positions encoding each gene. Significant intersections of genes that lost diversity during treatment were determined using the SuperExactTest (27) with default settings.

### Data availability.

All sequence read data generated for this research have been deposited in the Sequence Read Archive at the National Center for Biotechnology Information under BioProject ID PRJNA860728.
