# Supplementary material for: Genome Capture Sequencing Selectively Enriches Bacterial DNA and Enables Genome-Wide Measurement of Intrastrain Genetic Diversity in Human Infections
Source: mBio. 2022 Sep 19;13(5):e01424-22. doi: 10.1128/mbio.01424-22 (PMC9601202; doi:10.1128/mbio.01424-22)
Supplement: TEXT S1 [file mbio.01424-22-s0001.docx]

**Text S1.** Extended methods, including protocols for whole genome probe preparation and for probe hybridization and enrichment.

**A. Protocol for Whole Genome Probe Preparation**

**Fragment genomic DNA with M220 Focused-ultrasonicator (Covaris)**

1. Program Covaris to shear DNA to an average of 150bp fragments. Store samples in LoBind tubes from here on. (This protocol assumes sample in 130ul of reagent in a Cavairs Snap-Cap microtube.)

| Treatment Time | Setpoint, °C | Temp Range, °C | Peak Power | Cyclers/Burst | Avg Power |
| --- | --- | --- | --- | --- | --- |
| 350 sec | 20.0 | 18.0 | 50.0 | 200 | 10.0 |

1. Maximum amount of DNA per Covaris well: 5ug max quantity (keep 130ul volume).
2. Quantitate with Qubit High Sensitivity Kit (Thermo Fisher Scientific) here to determine concentration.

**Repair DNA fragments with NEBNext FFPE DNA Repair Mix (New England BioLabs)**

At this point transfer to a 96 or 48 well format.

1. Divide samples into 53.5ul aliquots in 96 well plates. Do not exceed 1ug per aliquot.
2. Add water to final aliquot if needed to reach a total volume of 53.5ul at a max of 1ug per aliquot.
3. Add remaining FFPE mix ingredients to each aliquot:

FFPE DNA Repair Buffer 10X = 6.5ul

NEBNext FFPE DNA Repair Mix = 2ul

Total Volume = 62ul

1. Mix by pipetting, followed by a quick spin.
2. Incubate at 20°C for 15 minutes.

**Clean up using Ampure XP beads (Beckman Coulter Life Sciences)**

1. Add 3X volume beads (186ul) to repair reaction. Pipette to mix thoroughly.

(e.g., 62ul total x 3 = 186ul vol of bead input)

1. Incubate at room temperature for 5 minutes
2. Place on magnet to clear beads ~5 minutes
3. Discard supernatant
4. Add 200ul 80% ethanol and allow to sit at room temp for 30 seconds
5. Discard supernatant
6. Repeat ethanol wash once
7. Discard supernatant and remove residual ethanol. Allow to air dry for 5 minutes on magnet
8. Remove from magnet and resuspend beads in 40ul H2O. Mix well. Inbuate for 5 minutes at room temp.
9. Clear beads on magnet for ~2 minutes
10. Transfer supernatant to fresh plate

**Quantitate using Qubit (Thermo Fisher Scientific)**

Aliquot fragmented and FFPE repaired DNA at 11pmol DNA ends per reaction (at 150bp fragments this means 450-500ng.)

SAFE STOPPING POINT: *Samples can be stored overnight at 4C.

**Dephosphorylation of sheared DNA using Shrimp Alkaline Phosphatase (rSAP) (New England BioLabs)**

1. Add dephosphylation reagents to sheared DNA for a total volume of 50ul per reaction:

Sheared DNA xul

Cutsmart Buffer (10X) 5ul

rSAP 2.5ul

H2O to 50ul xul

1. Incubate at 37°C for 2 hours followed by 65°C heat kill for 15 minutes.

**Biotinylation using Terminal Transferase (New England BioLabs) and Biotin-11-ddATP (Perkin Elmer)**

1. To the reaction add reagents for Biotinylation

Dilute Biotin 1:100 in water (0.5ul +49.5ul H2O)

CoCl2 (2.4mM) 5ul

Biotin- 11- ddATP (diluted) 1ul

Terminal Transferase 0.5ul

1. Incubate for 1.5 hours at 37°C. Heat inactivation at 70°C for 10 minutes.
2. Leave at 4°C overnight in the PCR block or in a refrigerator.

STOPPING POINT: *Samples can be stored overnight at 4°C before or after Monarch Kit Clean up.

**Remove unbound biotin with Monarch PCR & DNA Cleanup Kit (New England BioLabs)**

1. Remove unbound biotin with New England Biolab’s “Monarch PCR and DNA Cleanup kit” per manufacturer’s protocol.
2. If preparing one probe type, pool reactions from each sample from biotinylation step. Each Monarch filter column can purify up to 5 ug. At the end of the cleanup, elute DNA in 100uL EB from each column.

**Purify with Dynabeads MyOne Streptavidin C1 (Thermo Fisher Scientific) to eliminate non-biotinylated strands**

1. Wash Dynabeads MyOne Streptavidin C1:
   - Aliquot 50ul Dynabeads per reaction into a 1.5ml LoBind Eppendorf tube.
     - (eg. for 3 reactions, add 150ul of beads)
   - Place beads on magnet until clear ~2 minutes
   - Discard supernatant
   - Wash 3X as follows:
     - Add 200uL 2X Binding and Wash Buffer for each reaction (eg: for 3 reactions, you will need to add 600ul of buffer)
     - Pipet to resuspend beads
     - Clear beads on magnet
     - Discard supernatant
   - After 3 washes, resuspend beads in 100uL 2X Binding and Wash Buffer per reaction (eg: for 3 reactions, you will need 300ul of buffer)
   - Aliquot 100ul of resuspended beads into separate 1.5mL LoBind tubes.
2. Add sample to the washed beads.
   - Gently pipet to resuspend the beads.
   - Incubate at RT for 30 minutes (not on the magnet). Tap or vortex tube to mix beads 5-6 times during incubation.
3. Make 0.15M NaOH and 0.1M NaOH for later steps. See recipes section below.
4. Wash beads 3X with 200ul **1x** Binding and Wash Buffer as described previously during 3X wash step. Binding of DNA is now complete.

**Dissociate any strands that are non-biotinylated from their biotinylated and bound complements**

- 1. Wash the DNA coated Dynabeads in 100ul 1 x SSC.
  2. Resuspend the beads in 50ul of freshly prepared 0.15M NaOH.
     1. Incubate at room temperature for 10 minutes.
  3. Put the tube in magnet stand for 1–2 minutes and if desired, transfer the supernatant to a new tube. The supernatant contains your non-biotinylated DNA strand.
  4. Wash the Dynabeads coated with biotinylated strand once each with:
     1. 100ul 0.1M NaOH
     2. 100ul of with **1x** Binding and Wash
     3. 100ul 1X TE buffer.
  5. Resuspend beads in 50uL Sigma water **OR** Formamide mix (see recipe section).

**Dissociate biotinylated DNA from beads (Formamide method is most efficient)**

1. Heat Method (water)
   1. Put samples in cold Thermocycler and allow to warm up to 70°C.
   2. Incubate at 70°C for 1 minute.
   3. Remove samples from Thermocycler and place on magnet.
   4. Transfer supernatant containing biotin labeled DNA to clean tube.
2. Formamide Method
   1. Incubate at 65°C for 5 minutes.
   2. Place on magnet to clear beads ~2 minutes.
   3. Transfer supernatant to clean tube.

**Remove formamide with Monarch PCR & DNA Cleanup Kit (New England BioLabs) (**Modified ssDNA method)

1. A starting sample volume of 50ul is recommended. For smaller samples, nuclease-free water can be used to adjust the volume.

2. Add 100ul DNA Cleanup Binding Buffer to the 50μl sample.

3. Add 300ul ethanol (≥ 95%). Mix well by pipetting up and down or flicking the tube. Do not vortex.

4. Insert column into collection tube, load sample onto column and close the cap. Spin for 1 minute, then discard flow-through.

5. Re-insert column into collection tube. Add 500ul DNA Wash Buffer and spin for 1 minute. Discard flow-through.

6. Repeat Step 5.

7. Transfer column to a new LoBind 1.5 ml microfuge tube. Use care to ensure that the tip of the column does not come into contact with the flow-through. If in doubt, re-spin for 1 minute to ensure traces of salt and ethanol are not carried over to the next step.

Add ≥ 20-25ul of DNA Elution Buffer to the center of the matrix. Wait for 1 minute, then spin for 1 minute to elute the DNA.

**Recipes:**

**5M NaCl (14.61g NaCl to 50ml water) - heat gently to dissolve**

**2X Binding and Wash Buffer Recipe** (makes 50ml)

10mM Tris-HCl (eg. Trizma hydrochloride)

1mM EDTA

2M NaCl

To a 50ml conical add: 20ml of 5M NaCl, 500ul 1M Tris-HCl, and 100ul of 0.5M EDTA. Bring to 50ml with ultrapure water. Filter through a 0.2um syringe filter to sterilize.

**1X Binding and Wash Buffer**

Dilute 2X buffer with equal volume water

**0.15M NaOH** = 15ul 10M NaOH + 985ul water

**0.1M NaOH** = 10ul NaOH + 990ul water

**Formamide** = 950ul formamide + 20ul (0.5M) EDTA + 30ul water. Aliquot and store in -20. Avoid freeze thaws.

**B. Protocol for Probe Hybridization and Enrichment**

The xGen Hybridization and Wash Kit (Integrated DNA Technologies) used in this protocol is designed for multiplexing the hybridization capture. The protocol can also be performed in individual tubes, if desired.

**Before you start**

Two thermal cyclers, set at different incubation temperatures, are used for hybrid capture in this protocol. Create the following PCR programs:

HYB program (lid set at 100°C)

95°C – 10 min

65°C – 4hr – 16 hrs*

65°C – Hold

*Duration of hybridization should be kept consistent for all samples within a project. For GC-rich or small panels (<1000 probes), longer hybridization times (up to 16hr) may improve performance.

WASH program (lid set at 70°C*)

It is critical to reduce the lid temperature to 70°C for the WASH program.

65°C - Hold

**Perform hybridization reaction**

1. Start with aliquoting approximately 500ng of libraries of interest in individual 1.5 ml Eppendorf tubes.
2. Create the Blocker Master Mix in a tube. Multiply by the number of samples and add a 10% overfill.

| **Blocker Master Mix component** | **Volume per reaction (ul)** |
| --- | --- |
| Human Cot DNA | 5 |
| xGen Blocking Oligos | 2 |

1. Vortex to mix well.
2. Add 7ul of the Blocker Master Mix to each tube containing 500ng of library.
3. Dry down the mixture in a SpeedVac system (65°C for 20 min or more depending on the library volume), ensure all the contents are dry.

*Safe stopping point. Store samples at room temperature overnight, or -20°C for longer.

1. Thaw all contents of the xGen Hybridization and Wash Kit to room temperature. Inspect the tube of 2X Hybridization Buffer for crystallization of salts. If crystals are present, heat the tube at 65°C, shaking intermittently.
2. Create the Hybridization Master Mix in a tube. Multiply by the number of samples and add a 10% overfill.

| **Hybridization Master Mix component** | **Volume per reaction (ul)** |
| --- | --- |
| xGen 2X Hybridization Buffer | 8.5 |
| xGen Hybridization Buffer Enhancer | 2.7 |
| xGen Lockdown Panel or custom probes | 5.8 |

1. Vortex or pipet the mix to mix well.
2. Add 17ul of the Hybridization Master Mix to each well of the tubes containing dried DNA.
3. Transfer contents from the tubes to individual wells of a 96 well Lo bind plate.
4. Securely seal the plate with a Microseal B seal (BioRad).
5. Incubate at least 5 min at room temperature.
6. Vortex the samples, making sure that they are completely mixed.
7. Briefly centrifuge the samples.
8. Place the plate on the thermal cycler and start the HYB program.

**Prepare buffers**

**Note:** Before preparing the buffers, remove the Dynabeads M-270 Streptavidin beads from storage at 4°C. Equilibrate the beads at room temperature at least 30 min before performing the washes.

1. Dilute the following xGen buffers to create 1X working solutions as follows, multiplying by the required number of samples and adding 10% overfill:

| **Component** | **Nuclease-Free Water (ul)** | **Buffer (ul)** | **Total (ul)** |
| --- | --- | --- | --- |
| xGen 2X Bead Wash Buffer | 150 | 150 | 300 |
| xGen 10X Wash Buffer 1 | 225 | 25 | 250 |
| xGen 10X Wash Buffer 2 | 135 | 15 | 150 |
| xGen 10X Wash Buffer 3 | 135 | 15 | 150 |
| xGen 10X Stringent Wash Buffer | 270 | 30 | 300 |

**Note:** If Wash Buffer 1 is cloudy, heat the bottle in a 65°C water bath to allow resuspension.

1. Swirl to mix. Vortex will cause frothing.
2. The 1X working solutions are stable at room temperature (15–25°C) for up to 4 weeks.
3. Use a fresh PCR plate. For 32 samples, as an example, aliquot and label the plate as follows:

- Columns 1–4: 110 μL of Wash buffer 1
- Columns 5–8: 160 μL of Stringent Wash Buffer
- Columns 9–12: 160 μL of Stringent Wash Buffer

1. Do not discard the remaining Wash Buffer 1. The remaining buffer is needed to perform the Room temperature washes later in the protocol.
2. Seal the buffer plate and set aside.
3. In a LoBind tube, make the Bead Resuspension Mix. Multiply by the number of samples and add a 10% overfill.

| **Bead Resuspension Mix component** | **Volume per reaction (ul)** |
| --- | --- |
| xGen 2X Hybridization Buffer | 8.5 |
| xGen Hybridization Buffer Enhancer | 2.7 |
| Nuclease-Free Water | 5.8 |

**Wash Streptavidin beads**

Only perform bead washes with beads that have equilibrated to room temperature.

1. Mix the beads thoroughly by vortexing for 15 sec.
2. Add 50ul of Streptavidin beads to a new PCR plate, filling a well for every sample being captured.
3. Add 100ul of Bead Wash Buffer from Prepare buffers, step 1 to each well, then gently pipet the mix 10 times.
4. Place the plate containing beads on a magnet and allow the beads to fully separate from the supernatant (approximately 1 min).
5. Remove and discard the clear supernatant, ensuring that the beads remain in the well.
6. Remove the plate containing beads from the magnet.
7. Perform the following wash:
   1. Add 100ul of Bead Wash Buffer to each well containing beads, then gently pipet the mix 10 times.
   2. Place the plate on the magnet for approximately 1 min, allowing beads to fully separate from the supernatant.
   3. Carefully remove and discard the clear supernatant.
8. Perform an additional wash by repeating step 7 (above) for a total of 2 washes.
9. Resuspend the beads in 17ul of Bead Resuspension Mix from Prepare buffers, step 4.
10. Mix thoroughly to ensure the beads are not left to dry in the well. If needed, briefly centrifuge the plate containing beads at 25 x *g* (400 rpm).

**Perform bead capture**

If any of the sample accidentally splashes onto the plate seal while vortexing in Perform bead capture, briefly and gently centrifuge the plate (10 sec at 25 x *g*).

1. Start the WASH program in the second thermal cycler to start warming the buffer plate prepared in Prepare buffers, step 2. Make sure the lid temperature is set to 70°C for the WASH program.
2. The buffer plate needs to warm up for at least 15 min. Recommend starting incubation at the same time as the bead capture.
3. After the hybridization incubation is complete, remove the sample plate from the thermal cycler.
4. Once the sample plate has been removed from the instrument, stop the HYB program.
5. Immediately after the HYB program is complete, start the WASH program. At this point, both thermal cyclers should be running the WASH program.
6. Using a multichannel pipette and fresh filter tips, transfer the fully homogenized beads to the samples.
7. Securely seal the sample plate.
8. Gently vortex the sample plate until it is fully mixed, being careful not to splash onto the plate seal.
9. Place the sample plate in the thermal cycler for 45 min. During incubation, remove the plate every 10–12 min to quickly and gently vortex.

*****It is safe to place the sample plate in the thermal cycler before the lid temperature has fully cooled to 70°C when starting the incubation.

**Perform washes**

Always keep the buffer plate on the thermal cycler during washes. Make sure to reseal the buffer plate in between washes. When performing the heated washes, keep the buffer plate on the thermal cycler to maintain its set temperature.

Heated washes

1. After 45 min, remove the sample plate from the thermal cycler.
2. With the buffer plate remaining in the thermal cycler, transfer 100ul of heated Wash Buffer 1 to each sample and pipet the mix 10 times, being careful to minimize bubble formation.
3. Reseal the buffer plate, then close the lid.
4. Place the sample plate on the magnet for 1 min. Remove the supernatant.
5. Remove the sample plate from the magnet, then add 150ul of heated Stringent Wash Buffer to each well containing a sample. Reseal the buffer plate, then close the lid.
6. Pipet the mix 10 times, being careful to minimize bubble formation. Always use fresh pipette tips for each well.
7. Securely seal the sample plate, then incubate for 5 min in the thermal cycler.
8. Place the sample plate on the magnet for 1 min, then remove the supernatant.
9. Remove the sample plate from the magnet, then add 150ul of heated Stringent Wash Buffer from the buffer plate to the sample plate.
10. Pipet the mix 10 times, being careful to minimize bubble formation. Securely seal the sample plate, then incubate for 5 min on the thermal cycler.
11. Place the sample plate on the magnet for 1 min.

Room temperature washes

To ensure that the beads remain fully resuspended, vigorously mix the samples during the room temperature washes.

1. Remove supernatant. Add 150ul of Wash Buffer 1.
2. Securely seal the sample plate with a fresh seal, then vortex at full-speed thoroughly, until fully resuspended. It is critical to use a new seal at this step to avoid the risk of contamination because there will be some bead splash on the seal.
3. Incubate for 2 min while alternating between vortexing for 30 sec and resting for 30 sec, to ensure the mixture remains homogenous.
4. Centrifuge the sample plate for 5 sec at 25 x *g* to avoid well-to-well contamination.
5. Place the sample plate on the magnet for 1 min, then remove and discard the seal.
6. Remove the supernatant, then remove the sample plate from the magnet.
7. Add 150ul of Wash Buffer 2, then securely seal the sample plate with a fresh seal and vortex thoroughly until fully resuspended.
8. Incubate for 2 min while alternating between vortexing for 30 sec and resting for 30 sec, to ensure the mixture remains homogenous.
9. After the incubation, briefly centrifuge the sample plate (5 sec at 25 x *g*).
10. After centrifuging, place the sample plate on the magnet for 1 min, then remove and discard the seal.
11. Remove the supernatant, then remove the sample plate from the magnet.
12. Add 150ul of Wash Buffer 3, then securely seal the sample plate with a fresh seal and vortex thoroughly until fully resuspended.
13. Incubate for 2 min while alternating between vortexing for 30 sec and resting for 30 sec, to ensure the mixture remains homogenous.
14. After the incubation, briefly centrifuge the sample plate (5 sec at 25 x *g*).
15. After centrifuging, place the sample plate on the magnet for 1 min, then remove and discard the seal.
16. Remove the supernatant.
17. With the sample plate still on the magnet, use fresh pipette tips to ensure
    that all residual Wash Buffer 3 has been removed, then remove the plate from the magnet.
18. Add 20ul of Nuclease-Free Water to each capture.
19. Pipet the mix 10 times to resuspend any beads stuck to the side of the well.
20. Do not discard the beads. Use the entire 20 μL of resuspended beads with captured DNA in Perform post-capture PCR.

**Perform post-capture PCR**

1. In a tube, prepare the Amplification Reaction Mix, multiplied by the number of samples on the plate and adding 10% overfill, as follows:

| **Amplification Reaction Mix component** | **Volume (ul)** |
| --- | --- |
| 2X KAPA HiFi HotStart ReadyMix | 25 |
| 10 μM Illumina P5 primer | 2.5 |
| 10 μM Illumina P7 primer | 2.5 |
| Beads with captured DNA from step. 20 | 20 |

1. Mix for a final reaction volume of 50ul.
2. Securely seal the sample plate, then pipette 10x to thoroughly mix the reaction.
3. Briefly centrifuge the plate.
4. Place the plate in a thermal cycler, and run the following program with the lid temperature set to 105°C:

| **Step** | **Number of cycles** | **Temperature (°C)** | **Time** |
| --- | --- | --- | --- |
| Polymerase activation | 1 | 98 | 45 sec |
| Denaturation | 10-12  *10 cycles for samples with 1% or more initial relative abundance of target DNA, 12 cycles for samples with less than 1% of target DNA) | 98 | 15 sec |
| Annealing |  | 60 | 30 sec |
| Extension |  | 72 | 30 sec |
| Final extension | 1 | 72 | 1 min |
| Hold | 1 | 4 | Hold |

*Optional stopping point. Amplified captures may be stored at 4°C overnight.

**Purify post-capture PCR fragments**

Ensure Ampure XP beads (Beckman Coulter Life Sciences) have been equilibrated to room temperature before proceeding.

1. Prepare 250ul of fresh 80% ethanol per sample, multiplied by the number of samples with a 10% overfill.
2. Add 75ul (1.5X volume) of Ampure XP beads to each amplified capture.
3. After adding the beads, pipet the mix thoroughly and incubate for 5–10 min.
4. Place the plate on the magnet until the supernatant is clear (2–5 min).
5. Remove the supernatant without disturbing the beads.
6. While keeping the plate on the magnet, add 125ul of 80% ethanol, then incubate for 1 min.
7. Remove the ethanol, then repeat another ethanol wash.
8. Allow the beads to air dry for 1–3 min. Do not overdry the beads.
9. Remove the sample plate from the magnet and elute in 22ul of Buffer EB, or equivalent (10 mM Tris-Cl, pH 8.5). Mix thoroughly.
10. Incubate for 5 min at room temperature.
11. Place the plate on a magnet until supernatant is clear (1–2 min).
12. Transfer 20ul of eluate to a fresh plate, making sure that no beads are carried over.

*Purified PCR fragments may be stored at -20°C for up to 1 week.

**Validate, quantify library and sequencing**

1. Measure the concentration of the captured library using Qubit HS assay kit.
2. Measure the average fragment length of the captured library on the Tape Station.
3. Calculate molarity and multiplex for sequencing on Illumina platform.

**Consumables**:

1. xGen Hybridization and Wash Kit (Integrated DNA Technologies), 96 rxn
2. Blocking oligos - xGen Universal Blockers for Nextera libraries —NXT Mix, 96 rxn
3. Human Cot DNA (part of the Hyb and Wash Kit)
4. Ampure XP beads (Beckman Coulter Life Sciences)
5. Dynabeads M-270 Streptavidin beads (part of the Hyb and Wash Kit)
6. Eppendorf twin.tec 96 Well LoBind PCR Plates (Fisher Scientific)
7. KAPA HiFi HotStart ReadyMix (Roche)
8. Microseal B PCR Plate Sealing Film (Bio-Rad)
9. Qubit dsDNA HS Assay Kit (ThermoFisher Scientific)
10. Eppendorf tubes- 1.5 ml
11. TapeStation HS screentape and buffers (Agilent)

**Equipment:**

1. SpeedVac (ThermoFisher Scientific)
2. Magnetic Stand-96 (ThermoFisher Scientific)
3. TapeStation (Agilent)
4. Two thermal cyclers (placed side by side for optimal efficiency)
