# Supplementary material for: Genome Capture Sequencing Selectively Enriches Bacterial DNA and Enables Genome-Wide Measurement of Intrastrain Genetic Diversity in Human Infections
Source: mBio. 2022 Sep 19;13(5):e01424-22. doi: 10.1128/mbio.01424-22 (PMC9601202; doi:10.1128/mbio.01424-22)
Supplement: FIG S1 [file mbio.01424-22-s0002.docx]

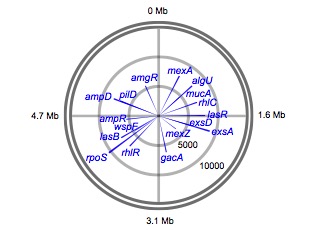


PAO1 chromosome

(Circular)

**Figure S1.** Representative circle plot for synthetic sample B (composed of 2% total *P. aeruginosa* and 98% human DNA) illustrating average read depth per million reads for each targeted gene with GenCap-Seq enrichment. Read depths are plotted as blue lines around the circular PAO1 reference genome (dark grey). Blue lines for four pairs of genes overlap due to close proximity in the genome (*algU/mucA*, *exsA/exsD*, *wspF/lasB*, and *ampD/pilD*). Approximate genome coordinates are indicated at vertical and horizontal lines (light grey), and concentric circles (light grey) indicate scale of average read depth per million reads sequenced.
