# Supplementary material for: Genome Capture Sequencing Selectively Enriches Bacterial DNA and Enables Genome-Wide Measurement of Intrastrain Genetic Diversity in Human Infections
Source: mBio. 2022 Sep 19;13(5):e01424-22. doi: 10.1128/mbio.01424-22 (PMC9601202; doi:10.1128/mbio.01424-22)
Supplement: TABLE S1 [file mbio.01424-22-s0003.docx]

**Table S1.** MetaPhlAn reported relative abundance of *E.coli* and *Escherichia* from shotgun sequencing of fecal samples and individual *E. coli* isolates.
