# Supplementary material for: Genome Capture Sequencing Selectively Enriches Bacterial DNA and Enables Genome-Wide Measurement of Intrastrain Genetic Diversity in Human Infections
Source: mBio. 2022 Sep 19;13(5):e01424-22. doi: 10.1128/mbio.01424-22 (PMC9601202; doi:10.1128/mbio.01424-22)
Supplement: TABLE S2 [file mbio.01424-22-s0004.docx]

**Table S2.** Coefficient of variation for individual measurements of 113 positions present in 20 core genes where single nucleotide polymorphisms distinguished the PAO1 and PACS2 *P. aeruginosa* strains.
