# Supplementary material for: Genome Capture Sequencing Selectively Enriches Bacterial DNA and Enables Genome-Wide Measurement of Intrastrain Genetic Diversity in Human Infections
Source: mBio. 2022 Sep 19;13(5):e01424-22. doi: 10.1128/mbio.01424-22 (PMC9601202; doi:10.1128/mbio.01424-22)
Supplement: TABLE S3 [file mbio.01424-22-s0005.docx]

**Table S3.** Coefficient of variation for measurements averaged across replicates of 113 positions present in 20 core genes where single nucleotide polymorphisms distinguished the PAO1 and PACS2 *P. aeruginosa* strains.
