# Supplementary material for: Genome Capture Sequencing Selectively Enriches Bacterial DNA and Enables Genome-Wide Measurement of Intrastrain Genetic Diversity in Human Infections
Source: mBio. 2022 Sep 19;13(5):e01424-22. doi: 10.1128/mbio.01424-22 (PMC9601202; doi:10.1128/mbio.01424-22)
Supplement: TABLE S4 [file mbio.01424-22-s0006.docx]

**Table S4.** Minor variant frequencies for 50 variable positions across 15 core genes for the 90:10 and 95:05 synthetic samples.
