# Supplementary material for: Genome Capture Sequencing Selectively Enriches Bacterial DNA and Enables Genome-Wide Measurement of Intrastrain Genetic Diversity in Human Infections
Source: mBio. 2022 Sep 19;13(5):e01424-22. doi: 10.1128/mbio.01424-22 (PMC9601202; doi:10.1128/mbio.01424-22)
Supplement: TABLE S5 [file mbio.01424-22-s0007.docx]

**Table S5.** Results of Population Multi-Locus sequence Typing (PopMLST), including locus type and frequency of locus type for each of six loci in *Pseudomonas aeruginosa* for 7 CF subjects before tobramycin treatment, indicating that all subjects were infected by a single *P. aeruginosa* strain.
