# Supplementary material for: Genome Capture Sequencing Selectively Enriches Bacterial DNA and Enables Genome-Wide Measurement of Intrastrain Genetic Diversity in Human Infections
Source: mBio. 2022 Sep 19;13(5):e01424-22. doi: 10.1128/mbio.01424-22 (PMC9601202; doi:10.1128/mbio.01424-22)
Supplement: TABLE S6 [file mbio.01424-22-s0008.docx]

**Table S6.** Average genome-wide π, change in average genome-wide π from “Off” to “On” tobramycin, and number of core genes and genes with non-zero π values for subject samples.

^1^ Sequence reads for patient samples were down sampled to 100X, except 205 (44X), 216 (79X), and 306 (65X) due to coverage limitations for one sample in each pair.

^2^ The number of genes common to both *P. aeruginosa* reference strain PAO1 (5677 total) and the subject’s isolate.
