# Supplementary material for: Genome Capture Sequencing Selectively Enriches Bacterial DNA and Enables Genome-Wide Measurement of Intrastrain Genetic Diversity in Human Infections
Source: mBio. 2022 Sep 19;13(5):e01424-22. doi: 10.1128/mbio.01424-22 (PMC9601202; doi:10.1128/mbio.01424-22)
Supplement: TABLE S7 [file mbio.01424-22-s0009.docx]

**Table S7.** Primers used to amplify genes for targeted GenCap-Seq probe construction.
